# Association of habitual intake of probiotic supplements and yogurt with characteristics of the gut microbiome in the multiethnic cohort adiposity phenotype study

**DOI:** 10.1017/gmb.2023.10

**Published:** 2023-07-13

**Authors:** Weiwen Chai, Gertraud Maskarinec, Unhee Lim, Carol J. Boushey, Lynne R. Wilkens, V. Wendy Setiawan, Loïc Le Marchand, Timothy W. Randolph, Isaac C. Jenkins, Johanna W. Lampe, Meredith A. J. Hullar

**Affiliations:** 1 Department of Nutrition and Health Sciences, University of Nebraska–Lincoln, Lincoln, NE, USA; 2 University of Hawai’i Cancer Center, Honolulu, HI, USA; 3 Department of Preventive Medicine, University of Southern California, Los Angeles, CA, USA; 4 Fred Hutchinson Cancer Center, Seattle, WA, USA

**Keywords:** probiotic supplements, yogurt, gut microbiome, multiethnic cohort

## Abstract

Consumption of probiotics and/or yogurt could be a solution for restoring the balance of the gut microbiota. This study examined associations of regular intake of probiotic supplements or yogurt with the gut microbiota among a diverse population of older adults (*N*=1,861; 60–72 years). Faecal microbial composition was obtained from 16S rRNA gene sequencing (V1–V3 region). General linear models were used to estimate the associations of probiotic supplement or yogurt intake with microbiome measures adjusting for covariates. Compared to non-yogurt consumers (*N*=1,023), regular yogurt consumers (≥once/week, *N*=818) had greater *Streptococcus* (β=0.29, *P*=0.0003) and lower *Odoribacter* (β=−0.33, *P*<0.0001) abundance. The directions of the above associations were consistent across the five ethnic groups but stronger among Japanese Americans (*Streptococcus:* β=0.56, *P*=0.0009; *Odoribacter:* β=−0.62, *P*=0.0005). Regular intake of probiotic supplements (*N*=175) was not associated with microbial characteristics (i.e., alpha diversity and the abundance of 152 bacteria genera). *Streptococcus* is one of the predominant bacteria genera in yogurt products, which may explain the positive association between yogurt consumption and *Streptococcus* abundance. Our analyses suggest that changes in *Odoribacter* were independent of changes in *Streptococcus* abundance. Future studies may investigate whether these microbial genera and their sub-level species mediate potential pathways between yogurt consumption and health.

## Introduction

The human microbiota composed of complex and diverse microbial communities, is now recognised as likely playing an important role in human health *(*Clemente et al., 2012; Miele et al., 2015; Thomas et al., 2017). Convincing evidence suggests aberrations in the gut microbiota composition and function are associated with several chronic disease conditions, such as obesity, type 2 diabetes, and inflammatory bowel diseases (le Chatelier et al., 2013; Tran et al., 2015; Cani and Everard, 2016; Choi et al., 2017). Thus, the relationships between gut microbiota and health highlight the importance of developing dietary strategies targeting the microbial community of the human gut.

Consumption of probiotics from supplements and foods could be key to restoring a healthy balance to the gut microbiota (Kim et al., 2019) and, thus, reducing the risk of developing chronic diseases. As defined by the Food and Agriculture Organization (FAO) of the United Nations and World Health Organization, probiotics are “live strains of strictly selected microorganisms which, when administered in adequate amounts, confer a health benefit on the host” (Food and Agriculture Organization [FAO], 2002). Previous research has documented the potential benefits of probiotics for weight-loss (Crovesy et al., 2017), glycaemic control (Ruan et al., 2015), and improved metabolic health profile (Aggarwal et al., 2013; Ivey et al., 2015). One large-scale cross-sectional analysis using data nationally representative of the U.S. found that ingesting probiotic supplements or yogurt was associated with a lower prevalence of obesity and hypertension, higher HDL cholesterol, and lower triglyceride levels (Lau et al., 2019).

Yogurt is a popular probiotic food because of its wide availability. Data from epidemiological studies and randomised clinical trials suggest yogurt improves metabolic health (Dumas et al., 2017) and reduces colorectal cancer risk (Pala et al., 2011). It is hypothesised that the potential beneficial effects of probiotic supplement or yogurt ingestion on human health may be mediated through a favourable modification of the gut microbiota. A recent study using an animal model reported gut microbiota and fermentation-derived branched chain hydroxy acids mediated health benefits of yogurt consumption in obese mice (Daniel et al., 2022). While a growing body of evidence has suggested yogurt consumption could potentially alter gut microbiota (Suzuki et al., 2017; Redondo-Useros et al., 2019; le Roy et al., 2022), the broad impact of yogurt consumption on the gut microbial community remains unclear. Due to high inter-person variability of gut microbiota (Derrien and van Hylckama Vlieg, 2015), larger studies, particularly those with a diverse population, are necessary to elucidate the role of dietary ingestion of probiotic products such as probiotic supplements and yogurt in shaping the ecosystem of gut microbiota and host heath. Thus, in the current analysis utilising data from adiposity phenotype study (APS), we assessed associations of habitual intake of probiotic supplements and yogurt with the gut microbiota in a subset of the multiethnic cohort (MEC) study with five ethnic groups. In addition, we examined whether these associations, if present, were consistent across ethnic groups. This study focuses on the above objectives as details of the APS design and its microbiome analysis (one of the main domains of APS) were described previously (Fu et al., 2016; Lim et al., 2019; Hullar et al., 2021).

## Methods

### Study population

The MEC is an ongoing, longitudinal study of more than 215,000 participants aged 45–75 years from five ethnic groups including Japanese American, white, Latino, African American, and Native Hawaiian. The cohort was assembled in Hawaii and Los Angeles from 1993 to 1996; details on recruitment and baseline information were reported previously (Kolonel et al., 2000). Briefly, participants were identified primarily through drivers’ license files, supplemented with voter registration lists in Hawaii and Medicare files in California, and completed a self-administered, 26-page questionnaire at cohort entry (1993–1996) assessing diet by semi-quantitative food frequency questionnaire (QFFQ), socio-demographic factors, anthropometric measures, medical history, family history of cancer, and lifestyle factors.

A subset of MEC members, aged 60–72 years as of January 2013 and living in the catchment area of the study clinics, were recruited for the APS conducted in 2013–2016, as described previously (Maskarinec et al., 2017; Lim et al., 2019). Participants were recruited within 60 sex/ethnicity/body mass index (BMI) strata with a participation rate of 25.6% after excluding the ineligible (*N* = 4,624) and too ill/deceased (*N* = 706) out of the 12,602 contacted MEC members. Blood and stool samples, anthropometric measures, questionnaire data, and MRI and DXA scans were obtained during the clinic visit. Individuals with the following characteristics were excluded from APS: current BMI outside 18.5–40 kg/m^2^, current or recent (<2 years) smoking, soft or metal implants (other than knee or hip replacement), or serious health conditions. Individuals who experienced weight change of >9 kg, or treatments or procedures with the potential to modify outcomes of interest were deferred for 6 months when their eligibility was reconsidered (Maskarinec et al., 2017; Lim et al., 2019). Institutional review boards at the University of Hawaii (CHS#17200) and University of Southern California (#HS-12–00623) approved the protocol. All participants provided written informed consent. The authors assert that all procedures contributing to this work comply with the ethical standards of the relevant national and institutional committees on human experimentation and with the Helsinki Declaration of 1975, as revised in 2008.

### Faecal sample collection and processing

Stool samples were collected at home using a collection tube containing 5 mL RNAlater (Fisher Scientific, Pittsburgh, PA, USA) and sterile 5 mm glass beads (Ambion, Waltham, MA, USA) to facilitate sample dispersion in RNAlater (Fu et al., 2016). Participants kept their samples in their freezers and brought them to the study clinic. Stool samples were stored in RNAlater at −80°C at study centres and shipped in bulk on dry ice to Fred Hutchinson Cancer Research Center. Stool samples were thawed and homogenised, and genomic DNA was extracted (Fu et al., 2016). Briefly, to optimise bacterial genomic DNA extraction, we did bead beating at 45 s (2×) each with samples placed on ice in between. Quality control samples, duplicate participant samples, and processing blanks were used to assess variation in library preparation and sequencing batches.

For paired-end sequencing of the V1–V3 region of the 16S rRNA gene, the 27F mod forward PCR primer sequence was 5°-AGRGTTNGATCMTGGCTYAG-3°. The 519 R reverse PCR primer sequence was 5°-GTNTTACNGCGGCKGCTG-3°. Three PCR (20 μl; 20 ng genomic DNA) reactions were performed using the HotStarTaq Plus Master Mix Kit (QIAGEN, Venlo, Netherlands) under the following conditions: 94°C for 3 min, followed by 28 cycles of 94°C for 30 s, 53°C for 40 s, and 72°C for 1 min, after which a final elongation step at 72°C for 5 min was performed. After amplification, quality of the PCR products was checked in 2% agarose gel. The three PCR products were pooled together in equal proportions based on their molecular weight and DNA concentrations. Sequencing was performed at Molecular Diagnostics, LLP (Shallowater, TX, USA) on the MiSeq using MiSeq Reagent Kit v3 following the manufacturer’s guidelines to obtain 2 × 300 bp paired-end reads (Illumina, San Diego, CA, USA).

### Microbiome bioinformatics data processing

To classify bacterial taxonomy, sequences were processed using QIIME v.1.8 (Caporaso et al., 2010) as previously described but updated in comparison to the previous study (Hullar et al., 2021). The filtering strategy for operational taxonomic units (OTUs) included parameters in QIIME to exclude low abundant sequences, singletons, and chimeras (Langille et al., 2013). The QIIME-processed sequences were aligned to the SILVA v132 database (release 111) as the reference library for 16S rRNA gene classification (Pruesse et al., 2007) using the PyNAST algorithm (Caporaso et al., 2010). Sequences were joined with the fastq-join method, using min_overlap = 15 and perc_max_diff = 12. After filtering sequences, the Nelson two-step method was used for OTU generation at 97% similarity with the SILVA database for closed reference OTU picking following the UCLUST algorithm (Edgar, 2010). Specifically, the sequences were classified using the matching SILVA taxonomy for OTUs found in the first step of the Nelson method, and using MOTHUR’s naïve Bayesian Classifier (Wang et al., 2007; Schloss et al., 2009) trained against the SILVA database for OTUs found in the second step. Sequences that did not align to the appropriate 16S rRNA gene region were removed.

Sequence counts in each sample for the phylum and genus level were generated without rarefaction. Sequence reads ranged at 9,831–178,452 (mean = 38,029, SD = 19,034; median = 34,008). Alpha diversity measures (phylogenetic diversity [Faith and Baker, 2007]; Shannon Index [Shannon and Weaver, 1998]; and Chao1 Index [Chao and Shen, 2003]) were calculated in QIIME based on the average of 10 subsamples with rarefaction to 10,000 sequences per sample.

### Probiotic supplements and yogurt intake

Information on dietary probiotic supplements and yogurt intake was primarily obtained from the Stool Collection Questionnaire (Fu et al., 2016) used in the APS. This questionnaire inquired on the sampling details (date, time, overnight freezing, and any collection problems), overall health (body weight and health concerns), past year history of antibiotic or antifungal medication use, gastric procedures, probiotic pill or laxative use, rural or urban childhood (birth to the age of 3 years) environment, and consumption of yogurt (see below for detailed description) and other probiotic foods (kefir, kimchi, home-made pickles, miso, tempeh, and natto), special diets or artificial sweeteners (Fu et al., 2016). As for yogurt or probiotic supplement intake, this questionnaire included one question on habitual yogurt consumption “*Have you in the past year consumed yogurt regularly (once a week or more)?”* and one question on habitual probiotic supplement intake “*Have you in the past year taken any probiotic pills regularly (once a week or more)?*” However, we were not able to determine how frequently participants consumed yogurt or probiotic supplements, such as “how many times per week” since this was not included in the stool collection questionnaire and APS QFFQ (see below).

Additional information on dietary intake was obtained from APS QFFQ derived from the original QFFQ. The development and calibration of the original QFFQ has been detailed previously (Stram et al., 2000). The original QFFQ was updated for APS to modify the food lists, amounts, and examples or names given for the food items without substantial change. APS QFFQ has information on how many servings (cup equivalents) of yogurt participants consumed per day, which was used to determine whether the information on daily yogurt servings from APS QFFQ was comparable to the information on habitual yogurt intake from the APS Stool Collection Questionnaire.

### Statistical analysis

All statistical modelling was conducted with SAS version 9.4 software (SAS Institute Inc., Cary, NC, USA). All genus and OTU variables had undergone ComBat-adjustment (Zhang et al., 2020) to correct values across laboratory batches, followed by centred log-ratio transformation (CLR) to account for their compositional nature as previously described (Hullar et al., 2021). To capture the association between yogurt or probiotic supplement consumption and the microbiome, we compared regular yogurt consumption (once per week or more) (Y) versus non-regular yogurt consumption (NY) and regular probiotic supplement (any kind, once per week or more) use (P) versus non-regular probiotic supplementation (NP) using linear models regressed outcome variables (alpha diversity, genera abundance, etc.) on P (with NP as the reference category) or Y (with NY as the reference category). The regression coefficients (β) in these models indicate the difference in the relative abundance of a gut microbiome component per category as compared to the reference category. We conducted sensitivity tests by comparing P versus neither P nor Y (NPY) and comparing Y versus NPY. Due to the small number of participants who consumed both yogurt and probiotic supplements, we did not assess the associations of consumption of both dietary items (probiotic supplements + yogurt) with microbiota. Bonferroni-corrected *p*-value of 0.05/152 = 0.00033 was applied to the analysis of 152 genera to maintain a nominal type-I error of 0.05 and reduce the likelihood of chance associations.

We also performed sub-analyses for associations of P or Y with select OTUs. We included OTUs belonging to the specific genera which are commonly found in probiotic supplements and/or yogurt (Kok and Hutkins, 2018), such as *Bifidobacterium, Lactobacillus,* and *Streptococcus* and to the genera for which significant associations were detected (*P* < 0.00033 after Bonferroni correction). In our study, among those who reported taking probiotic supplements regularly, most were taking *Bifidobacterium* and/or *Lactobacillus* supplements (73%), supporting our OTU selection criteria mentioned above. All models were adjusted for ethnicity (African American, Japanese American, Latino, Native Hawaiian, or white), sex, age at stool collection, BMI at stool collection, physical activity (hour(s) of moderate/vigorous activity per day), smoking status (never or former), having antibiotic treatment(s) within the last year, total energy intake (log-transformed), and dietary fibre intake (log-transformed). The above analyses were repeatedly stratified by ethnicity. Additionally, we repeated analyses further adjusting for other parameters relevant to metabolic health such as viscera to subcutaneous fat ratio and serum insulin, glucose, total, HDL and LDL cholesterol, and triglycerides levels. We also assessed the associations between daily servings of yogurt intake from the APS QFFQ with microbiota variables for confirmation of the effects of yogurt on the gut microbiota. Since 21.6% of the participants had been treated with antibiotics during the past year, we also repeated the main analyses by excluding those previously treated with antibiotics.

## Results

A total of 1,861 generally healthy, older participants were included in the analyses. About 9.4% reported taking probiotic supplements (P) and 44.0% reported consuming yogurt regularly (Y). In addition, 6.0% reported intake of both dietary items, while 51.1% reported neither (NPY). Missing information on probiotic supplements and yogurt intake were 1.8% (*N* = 34) and 1.1% (*N* = 20), respectively. The mean age of study participants was 69.2 ± 2.7 years, with men (49.6%) and women (50.4%) evenly distributed. Of these, 17.0% were African Americans, 23.3% were Japanese Americans, 21.1% were Latinos, 16.5% were Native Hawaiians, and 22.1% were whites. The mean BMI was 28.0 ± 4.8 kg/m^2^. Among those taking antibiotics during the past year (21.6%), 53.9% consumed P or Y while the proportion for P or Y intake was 45.5% among non-antibiotic users. Women were more likely to consume P or Y compared to men (*P <* 0.0001). Dietary fibre intake was higher for those consuming P or Y than NPY (*P* < 0.0001). In addition, although there was no difference in BMI between P or Y and NPY, visceral to subcutaneous fat ratio and serum insulin and glucose levels were lower for P or Y compared to NPY (*P* < 0.05). P or Y had higher HDL but also higher total and LDL cholesterol levels relative to NPY (*P* < 0.05; Table 1). In the APS QFFQ, regular Y consumers reported higher daily yogurt consumption (0.31 ± 0.32 serving/day [≈75.9 ± 78.4 g/day]) than NY users (0.04 ± 0.06 serving/day [≈9.8 ± 14.7 g/day]; *P* < 0.0001).

**Table 1.** Characteristics of APS participants by status of intake of probiotic supplements and/or yogurt

	All	P or Y	NPY	*P* ^a^
*N* ^b^	1,861	882	951	
Age at stool collection (year)	69.2 ± 2.7	69.1 ± 2.7	69.3 ± 2.8	0.16
Sex, *N* (%)				<0.0001
Male	923 (49.6)	324 (37.2)	583 (61.3)	
Female	938 (50.4)	548 (62.8)	368 (38.7)	
Ethnicity, *N* (%)				<0.0001
African American	317 (17.0)	142 (16.3)	169 (17.8)	
Japanese American	434 (23.3)	181 (20.8)	253 (26.6)	
Latino	392 (21.1)	190 (21.8)	181 (19.0)	
Native Hawaiian	307 (16.5)	126 (14.5)	174 (18.3)	
White	411 (22.1)	233 (26.7)	174 (18.3)	
BMI at stool collection (kg/m^2^)	28.0 ± 4.8	27.9 ± 4.9	27.9 ± 4.8	0.86
Metabolic health parameters				
Ratio of visceral to subcutaneous fat	0.88 ± 0.52	0.79 ± 0.48	0.96 ± 0.52	<0.0001
Insulin (mg/dL)	7.2 ± 4.8	6.8 ± 4.5	7.4 ± 5.2	0.01
Glucose (mg/dL)	108.5 ± 31.1	105.7 ± 27.0	110.7 ± 33.5	0.0004
HDL cholesterol (mg/dL)	58.4 ± 24.8	59.7 ± 24.7	57.4 ± 24.6	0.045
LDL cholesterol (mg/dL)	127.2 ± 50.0	130.3 ± 51.5	123.6 ± 48.6	0.005
Total cholesterol (mg/dL)	207.9 ± 55.5	212.1 ± 56.7	203.5 ± 54.4	0.001
Triglycerides (mg/dL)	112.2 ± 59.1	111.4 ± 60.7	112.4 ± 57.8	0.73
Smoking status, *N* (%)				0.001
Never	1,112 (60.7)	569 (65.3)	551 (57.9)	
Former	719 (39.3)	303 (34.7)	400 (42.1)	
Current	0 (0.0)	0 (0.0)	0 (0.0)	
Moderate/vigorous activities (hour/day)	1.5 ± 1.4	1.5 ± 1.4	1.5 ± 1.4	0.96
Alcohol intake (g/day)	7.8 ± 17.1	7.1 ± 16.4	8.5 ± 17.7	0.09
Antibiotic use, *N* (%)	401 (21.6)	216 (24.8)	182 (19.4)	0.01
Total energy intake (kcal/day)	1,882 ± 946	1,907 ± 966	1,853 ± 922	0.23
Dietary fibre intake (g/day)	23.5 ± 14.3	25.1 ± 14.3	22.0 ± 14.0	<0.0001

Abbreviations: APS, adiposity phenotype study; NPY, consuming neither probiotic supplements nor yogurt regularly; P, regularly consuming probiotic supplements; Y, regularly consuming yogurt.

a *P*-values for differences between participants categorised as P or Y and participants categorised as NPY using *t-*test for continuous variables and chi-square test for categorical variables.

b In total, 34 participants had missing values of P; 20 participants had missing values of Y; and 28 participants had missing values of both P and Y.

The results of associations of P (vs. NP) and Y (vs. NY) with gut microbiota composition (152 analysed bacteria genera) are presented in Supplementary Table 1. Table 2 summarises the key results. Compared to NY, Y had a significantly higher abundance of *Streptococcus* genus (β = 0.29, *P* = 0.0003) and a lower abundance of *Odoribacter* (β = −0.33, *P* < 0.0001). No significant associations were found between P and abundance of 152 bacteria genera studied, including *Bifidobacterium* and *Lactobacillus* which are commonly consumed as probiotic supplements. Additionally, Y or P did not affect α diversity measures. For OTUs, one *Streptococcus* related OTU (*Streptococcus; uncultured bacterium*) was positively associated with Y versus NY (β = 0.40, *P* < 0.0001). Daily servings of yogurt consumption from APS QFFQ were also positively associated with the relative abundance of *Streptococcus* (β = 0.63, *P* < 0.0001) and the aforementioned OTU of *Streptococcus* (β = 0.93, *P* < 0.0001) as well as inversely associated with *Odoribacter* abundance (β = −0.41, *P* = 0.003). However, the latter did not reach statistical significance after Bonferroni correction. Sensitivity tests by comparing P versus NPY and Y versus NPY did not change the results significantly. Results did not change materially after further adjusting for parameters relevant to metabolic health (viscera to subcutaneous fat ratio, insulin, glucose, total, HDL and LDL cholesterol, and triglycerides). In addition, results did not change significantly after repeating the primary analyses with the exclusion of participants who were treated with antibiotics during the past year. After performing the analyses across all 152 bacteria genera (Supplementary Table 1), we focused on *Streptococcus* and *Odoribacter* since these two bacteria genera showed significant associations with yogurt consumption. We selected *Bifidobacterium* and *Lactobacillus* genera because these are commonly found in probiotic supplements and commercial yogurt products.

**Table 2.** Associations of probiotic supplement use and yogurt consumption with selected gut microbiota variables in APS

Measures	Yogurt^a^ (serving/day) *N* = 1,861^b^	P (vs. NP) *N* = 175 (vs. 1,652)^b^	Y (vs. NY) *N* = 818 (vs. 1,023)^b^
	β (*P*)^c^^,^^d^	β (*P*)^c^^,^^d^	β (*P*)^c^^,^^d^
Genera common in probiotic supplements and/or yogurt			
*Bifidobacterium*	−0.07 (0.59)	−0.08 (0.47)	−0.02 (0.78)
*Lactobacillus*	−0.04 (0.66)	−0.06 (0.47)	−0.01 (0.81)
*Streptococcus*	**0.63 (<0.0001** **)**	0.13 (0.29)	**0.29 (0.0003)**
Other genera			
*Odoribacter*	−0.41 (0.003)	−0.07 (0.55)	**−0.33 (<0.0001)**
OTUs			
*Streptococcus; uncultured_bacterium*	**0.93 (<0.0001)**	0.17 (0.21)	**0.40 (<0.0001)**

Abbreviations: APS, adiposity phenotype study; NP, not consuming probiotic supplements regularly; NY, not consuming yogurt regularly; P, regularly consuming probiotic supplements; Y, regularly consuming yogurt.

a Daily yogurt consumed (serving/day) from APS QFFQ.

b A total of 1,861 participants were included in the analyses: P (*N* = 175), NP (*N* = 1,652), 34 participants had missing data of P or NP; Y (*N* = 818), NY (*N* = 1,023), 20 participants had missing data of Y or NY.

c Beta coefficient (β) and *P*-value were estimated using proc GLM adjusting for age, sex, ethnicity, antibiotic intake, smoking status, daily moderate/vigorous physical activity hours, body mass index, dietary fibre intake, and total calories.

d Bonferroni-corrected *p*-value of 0.05/152 = 0.00033 was applied. (Bolded values indicate statistical significance after Bonferroni-correction, p-value < 0.00033).

**Figure 1.**
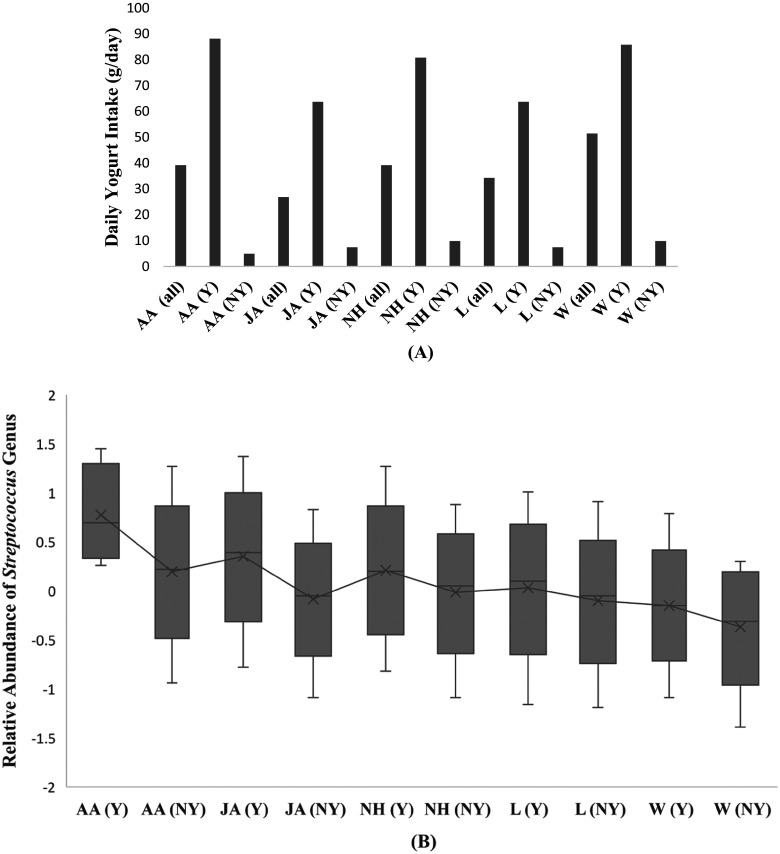
(A) Daily yogurt intake (g/day) for all participants (all), regular yogurt consumers (Y) and non-yogurt consumers (NY) by five ethnic groups. (B) Relative abundance of *Streptococcus* genus for regular yogurt consumers (Y) and non-yogurt consumers (NY) by five ethnic groups. AA, African American; JA, Japanese American; NH, Native Hawaiian; L, Latino; W, White. The bottom and top lines of the box represent lower and upper ends of 95% confidence interval of the mean, respectively. The middle line of the box and “X” marker represent mean value. The lowest and highest points of each graph represent 25 and 75 percentiles, respectively. *P*-values for associations of regular yogurt consumption with relative abundance of *Streptococcus* genus for the five ethic groups were: AA: *P* = 0.13; JA: *P* = 0.0009; NH: *P* = 0.32; L: *P* = 0.38; W: *P* = 0.32.

Results of associations of habitual yogurt intake with the abundance of 152 bacteria genera and *Streptococcus* OTU (uncultured) by five ethnic groups are presented in Supplementary Table 2. For *Streptococcus* and *Odoribacter* as well as one uncultured *Streptococcus* OTU, the associations with Y users (vs. NY) were stronger among Japanese Americans (*Streptococcus:* β = 0.56, *P* = 0.0009; *Odoribacter:* β = −0.62, *P* = 0.0004; *Streptococcus* OTU [uncultured]: β = 0.70, *P* < 0.0001) than in other ethnic groups. However, the directions of the associations were consistent across the five ethnic groups. Based on the APS QFFQ, white participants had the highest daily yogurt consumption (0.21 ± 0.26 serving/day [≈51.5 ± 63.7 g/day]) and Japanese Americans had the lowest (0.11 ± 0.19 serving/day [≈27.0 ± 46.5 g/day]) among the five ethic groups. The remaining ethnic groups, African Americans (0.16 ± 0.32 serving/day [≈39.2 ± 78.4 g/day]), Native Hawaiians (0.16 ± 0.31 serving/day [≈39.2 ± 75.9 g/day]), and Latinos (0.14 ± 0.23 serving/day [34.3 ± 56.3 g/day]), had similar daily yogurt intake.Figure 1A,B shows daily yogurt intake and relative abundance of *Streptococcus* genus by status of yogurt consumption (Y vs. NY) across the five ethnic groups. The daily yogurt consumptions were similar among the ethnic groups for NY. For Y, despite their highest daily yogurt intake, whites had the lowest abundance of *Streptococcus* genus (−0.15 ± 0.09). The difference in *Streptococcus* genus abundance between Y and NY was most prominent among Japanese Americans (Y: 0.39 ± 1.54 vs. NY: −0.05 ± 1.60, *P* = 0.0009).

## Discussion

This study included a subset of MEC volunteers from a multiethnic, older study population and observed that, compared to those who did not consume yogurt regularly, habitual yogurt intake was positively associated with the abundance of *Streptococcus* and inversely associated with the abundance of *Odoribacter.* These associations were stronger among Japanese Americans than in other ethnic groups; however, the directions were consistent across the five ethnic groups.

Emerging evidence suggests that the microorganisms associated with fermentation, along with probiotics added to fermented foods may contribute to human health (Marco et al., 2017; Kok and Hutkins, 2018). Yogurt is made with a culture containing strains of *Streptococcus thermophilus* and *Lactobacillus delbrueckii.* In addition, commercial yogurt products are commonly supplemented with probiotic bacteria, such as *Bifidobacterium* and *Lactobacillus* strains, for added benefits (Kok and Hutkins, 2018). Previous studies found a diet rich in yogurt was associated with a reduced risk of metabolic syndrome (Sonestedt et al., 2011) and colorectal cancer (Pala et al., 2011). Evidence from randomised clinical trials suggests that consumption of yogurt containing *Lactobacillus bulgaricus* and *Streptococcus thermophilus* had either favourable or neutral effects on markers of metabolic risk compared to the control treatment (Dumas et al., 2017). Additionally, several randomised trials show that yogurts with added probiotic bacterial strains were more effective than conventional yogurts in improving blood glucose (Ejtahed et al., 2012) and insulin resistance (Asemi et al., 2013; Madjd et al., 2016). Furthermore*, Streptococcus thermophilus,* used in yogurt production and other fermented milk products along with *Bifidobacterium* strains, has been shown to protect the gastrointestinal epithelium from *Escherichia coli*, improve somatic growth and reduce the severity and duration of acute diarrhoea in infants (Thibault et al., 2004; Corrêa et al., 2005). More recent research reported *Streptococcus thermophilus* inhibited colorectal tumorigenesis in animal models (Li et al., 2021).

In the current study, we found that habitually consuming yogurt (at least once a week) was positively associated with the relative abundance of *Streptococcus.* Additionally, the results of sub-analyses of OTUs indicated that intake of yogurt was also associated with one of the *Streptococcus* strains, although we were not able to identify the specific strain because of its uncultured status. Our findings were consistent with two recent studies that reported yogurt consumption was related to higher levels of yogurt starter *Streptococcus thermophilus* in 260 participants aged between 25 and 50 years (Redondo-Useros et al., 2019) and in 1,103 older adult participants from LifeLines-DEEP cohort (le Roy et al., 2022). A cross-sectional study conducted in 293 young adults in Japan reported that yogurt and fermented dairy product consumption showed positive associations with *Lactobacillus* and *Lactobacillus gasseri* subgroup and negative associations with *Staphylococcus* in both male and female subjects (Suzuki et al., 2017). Additionally, an increase in *Bifidobacterium* species was observed in *Bifidobacterium*-containing fermented milk consumers in 260 adult participants (Redondo-Useros et al., 2019). A positive association between the frequency of a specific fermented milk product consumption and gut microbiota diversity was also detected in more than 1,000 adult subjects (Zhernakova et al., 2016). However, we did not observe associations of yogurt intake with other two common genera, *Lactobacillus* (found in conventional yogurt and/or commercial yogurt products) and *Bifidobacterium* (the strains of *Bifidobacterium* are often added to commercial yogurt products). We also did not observe associations between regular probiotic supplement or yogurt intake with higher alpha diversity in our study.

We did not observe associations of probiotic supplement use with the 152 studied genera in the study, including *Lactobacillus* and *Bifidobacterium*, frequently found in probiotic supplements. In our study, compared to yogurt consumption (43.9%), considerably fewer participants reported taking probiotic supplements regularly (9.4%), which may, in part explain the null associations between probiotic supplement use and microbiome genera. In addition, due to the small number of participants who reported consuming both yogurt and probiotic supplements regularly (*N* = 111, 6%), we were not able to examine the synergic effects of yogurt intake and use of probiotic supplements on gut microbiota.

The current study also found that regular yogurt intake was inversely associated with *Odoribacter* abundance. It has been suggested that the potential beneficial effects of *Odoribacter* as part of a healthy, balanced human gut microbiota are primarily attributed to its capacity to produce short-chain fatty acids (SCFAs) (Hiippala et al., 2020). However, the role of *Odoribacter* and SCFAs in metabolic health remains unclear. One study reported *Odoribacter* abundance was inversed associated with systolic blood pressure in obese and overweight pregnant women, suggesting the possible influence on host blood pressure by this SCFA-producing microbiome genus (Gomez-Arango et al., 2016). Several studies also found that *Odoribacter* may benefit metabolic health coupled with other SCFA-producing genera, such as *Akkermansia* (Brahe et al., 2015; Etxeberria et al., 2017; Lim et al., 2017; Lai et al., 2018). In contrast, one study revealed that individuals with hypercholesterolemia were characterised by a higher prevalence of *Odoribacter* compared to those without hypercholesterolemia (Granado-Serrano et al., 2019). Another study observed a positive correlation between abundance of *Odoribacter* and fasting plasma glucose (Bellikci-Koyu et al., 2019). The inverse association between yogurt intake and *Odoribacter* observed in the current study should be confirmed by future investigations. We are unsure whether the lower abundance of *Odoribacter* with yogurt consumption was directly attributable to yogurt intake or mediated by the change of other microbial contents in the gut. Besides *Streptococcus* and *Odoribacter*, we did not observe significant associations of yogurt intake with other microbiota genera. We additionally adjusted for *Streptococcus* in the regression model between yogurt consumption and *Odoribacter* abundance, and results did not change significantly. This suggests the independence of *Streptococcus* and *Odoribacter* in relation to yogurt consumption. Thus, the relationships between yogurt consumption, *Odoribacter,* and metabolic health need to be further investigated in the context of the dynamic gut microbiota community.

In previous reports, gut microbiota composition varied by ethnicity, diet, and lifestyle (de Filippo et al., 2010; Kau et al., 2011; Wu et al., 2011; Chen et al., 2016). One study compared gut microbiota between Japanese Americans and native Japanese and found a lower *Odoribacter* abundance in Japanese Americans, possibly due to adopting more Westernised lifestyles (Yamashita et al., 2019). In the current study, we observed a stronger association of yogurt consumption with *Streptococcus* (positive association) and *Odoribacter* (inverse association) among Japanese Americans than in other ethnic groups. However, the directions of the above associations were consistent across the ethnic groups in the study. Hullar et al. (2021) assessed gut microbiome with non-alcoholic fatty liver diseases (NAFLDs) in the MEC APS (the same study population used in the current study). They found 69 genera were significantly associated with NAFLD in at least one ethnic group but no single genus was significantly associated with NAFLD across all ethnicities (Hullar et al., 2021), suggesting that ethnic-specific microbial composition and pathophysiologic pathways may provide the basis for targeted therapies for NAFLD and other metabolic diseases. The associations of habitual yogurt intake with *Streptococcus* genus in ethnic-specific patterns observed in our study are of interest. Generally speaking, *Streptococcus* abundance was greater in yogurt consumers relative to non-consumers across the five ethnic groups; however, the difference was more significant among Japanese Americans. Furthermore, despite the highest percentage of regular yogurt consumers (53.7%) and the highest daily yogurt intake observed in whites compared to other ethnic groups, whites had the lowest relative abundance of *Streptococcus* genus even for the regular yogurt consumers. Thus, the above results suggest that the relation between yogurt consumption and greater abundance of *Streptococcus* genus appeared to be ethnic-specific, which may provide new insight into understanding the potential pathway of dietary yogurt intake-alteration of gut microbiome-improvement of metabolic health once replicated in other studies.

Our study is the first large-scale, population-based study with multiple ethnic groups that examined relationships between probiotic supplement or yogurt intake and gut microbiota. There are several limitations of the current study. The small number of participants who reported taking probiotic supplements may have limited our statistical power to detect the potential associations of probiotic supplement intake with the gut microbiota. We only had one stool sample per participant with which to characterise an individual’s microbiome; however, previous studies have shown that a single assessment adequately captures the interindividual variation (Claesson et al., 2011; Fu et al., 2019). In addition, the stool collection questionnaire and APS QFFQ did not specifically ask participants about how often they consumed yogurt or probiotic supplements, for example “how many times per week”. Therefore, we may not wholly capture participants’ dietary habits related to yogurt or probiotic supplement intake. However, participants’ responses to the habitual yogurt consumption question were comparable to the data on their daily servings of yogurt intake from APS QFFQ, and similar results were observed for associations of daily yogurt servings from APS QFFQ with microbial variables (*Streptococcus, Odoribacter*, and *Streptococcus* OTU). Lastly, the study was limited to microbiome analysis which relied only on 16S rRNA gene sequencing.

In conclusion, the results from the current study suggest that regular consumption of yogurt was related to a greater abundance of *Streptococcus* and lower abundance of *Odoribacter* among generally healthy, older adults. The associations of yogurt with *Streptococcus* and *Odoribacter* were stronger in Japanese Americans than in other ethnic groups; however, the directions of the associations were consistent across ethnicity. No associations were found between probiotic supplement intake and gut microbial variables; Alpha diversity was not affected by yogurt or probiotic supplement consumption. Future studies need to confirm the current results and further identify the specific species of *Streptococcus* that was associated with yogurt intake. In addition, investigating whether microbial genera such as *Streptococcus* and *Odoribacter* and their sub-level species could mediate the potential pathway between dietary yogurt consumption and human health, in terms of improving metabolic outcomes is warranted.

## Data Availability

The data underlying this study cannot be made publicly available because they contain patient identifying information. Data are available from the multiethnic cohort study (http://www.uhcancercenter.org/research/the-multiethnic-cohort-study-mec/data-sharing-mec) for researchers who meet the criteria for access to confidential data. All sequencing data are publicly available at the NIH SRA (https://www.ncbi.nlm.nih.gov/sra) under accessions PRJNA804208, PRJNA629344, and PRJNA723466.

## Supplementary material

For supplementary material accompanying this paper visit https://doi.org/10.1017/gmb.2023.10. click here to view supplementary material
