# Supplementary material for: Association of habitual intake of probiotic supplements and yogurt with characteristics of the gut microbiome in the multiethnic cohort adiposity phenotype study
Source: Gut Microbiome (Camb). 2023 Jul 13;4:e14. doi: 10.1017/gmb.2023.10 (PMC10927272; doi:10.1017/gmb.2023.10)
Supplement: Supplementary file 1 [file S2632289723000105sup001.docx]

**Supplemental Table 1: Association of regular probiotic supplement use and yogurt consumption with microbiota composition (152 analyzed genera) in the Adiposity Phenotype Study**

| **Genera** | **P (vs. NP)**  N=175 (vs. 1,652)^a^ | **Y (vs. NY)**  N=818 (vs. 1,023)^a^ |
| --- | --- | --- |
|  | β (*P*)^bc^ | β (*P*)^bc^ |
| *Firmicutes; Negativicutes; Selenomonadales; Acidaminococcaceae; Acidaminococcus* | -0.06 (0.31) | 0.01 (0.69) |
| *Actinobacteria; Coriobacteriia; Coriobacteriales; Eggerthellaceae; Adlercreutzia* | 0.15 (0.08) | 0.002 (0.97) |
| *Firmicutes; Clostridia; Clostridiales; Lachnospiraceae; Agathobacter* | 0.04 (0.62) | 0.04 (0.40) |
| *Firmicutes; Clostridia; Clostridiales; Lachnospiraceae; Agathobacter* | 0.13 (0.16) | -0.09 (0.11) |
| *Verrucomicrobia; Verrucomicrobiae; Verrucomicrobiales; Akkermansiaceae; Akkermansia* | 0.13 (0.42) | -0.01 (0.89) |
| *Bacteroidetes; Bacteroidia; Bacteroidales; Rikenellaceae; Alistipes* | -0.10 (0.38) | -0.16 (0.02) |
| *Bacteroidetes; Bacteroidia; Bacteroidales; Prevotellaceae; Alloprevotella* | -0.005 (0.96) | -0.03 (0.63) |
| *Firmicutes; Clostridia; Clostridiales; Lachnospiraceae; Anaerostipes* | 0.03 (0.72) | 0.07 (0.14) |
| *Firmicutes; Clostridia; Clostridiales; Ruminococcaceae; Anaerotruncus* | -0.04 (0.63) | -0.01 (0.84) |
| *Bacteroidetes; Bacteroidia; Bacteroidales; Bacteroidales_RF16_group; uncultured_bacterium* | -0.29 (0.02) | 0.02 (0.78) |
| *Bacteroidetes; Bacteroidia; Bacteroidales; Other; Other* | 0.15 (0.11) | -0.02 (0.72) |
| *Bacteroidetes; Bacteroidia; Bacteroidales; Bacteroidaceae; Bacteroides* | 0.12 (0.05) | -0.05 (0.19) |
| *Firmicutes; Clostridia; Clostridiales; Lachnospiraceae; [Bacteroides]_pectinophilus_group* | -0.03 (0.77) | -0.002 (0.98) |
| *Bacteroidetes; Bacteroidia; Bacteroidales; Barnesiellaceae; Barnesiella* | 0.04 (0.77) | -0.03 (0.75) |
| *Bacteroidetes; Bacteroidia; Bacteroidales; Barnesiellaceae; uncultured* | 0.05 (0.54) | -0.02 (0.64) |
| *Actinobacteria; Actinobacteria; Bifidobacteriales; Bifidobacteriaceae; Bifidobacterium* | -0.08 (0.47) | -0.02 (0.83) |
| *Proteobacteria; Deltaproteobacteria; Desulfovibrionales; Desulfovibrionaceae; Bilophila* | -0.17 (0.048) | -0.03 (0.61) |
| *Firmicutes; Clostridia; Clostridiales; Lachnospiraceae; Blautia* | 0.11 (0.04) | -0.006 (0.86) |
| *Firmicutes; Clostridia; Clostridiales; Ruminococcaceae; Butyricicoccus* | -0.005 (0.94) | -0.008 (0.87) |
| *Bacteroidetes; Bacteroidia; Bacteroidales; Marinifilaceae; Butyricimonas* | -0.05 (0.59) | -0.007 (0.89) |
| *Firmicutes; Clostridia; Clostridiales; Lachnospiraceae; Butyrivibrio* | -0.09 (0.39) | 0.08 (0.21) |
| *Firmicutes; Clostridia; Clostridiales; Ruminococcaceae; CAG-352* | -0.003 (0.98) | 0.04 (0.64) |
| *Firmicutes; Erysipelotrichia; Erysipelotrichales; Erysipelotrichaceae; Candidatus_Stoquefichus* | -0.11 (0.19) | -0.003 (0.95) |
| *Firmicutes; Erysipelotrichia; Erysipelotrichales; Erysipelotrichaceae; Catenibacterium* | -0.14 (0.33) | -0.14 (0.11) |
| *Taxon=Bacteria; Firmicutes; Clostridia; Clostridiales;Christensenellaceae; Christensenellaceae_R-7_group* | -0.10 (0.47) | 0.02 (0.84) |
| *Synergistetes; Synergistia; Synergistales; Synergistaceae; Cloacibacillus* | 0.09 (0.28) | 0.05 (0.34) |
| *Firmicutes; Clostridia; Clostridiales;Other;Other* | 0.005 (0.92) | 0.004 (0.89) |
| *Firmicutes; Clostridia; Clostridiales; Clostridiales_vadinBB60_group; uncultured_bacterium* | -0.10 (0.36) | 0.07 (0.31) |
| *Firmicutes; Clostridia; Clostridiales; Clostridiales_vadinBB60_group; uncultured_organism* | -0.12 (0.11) | 0.02 (0.66) |
| *Firmicutes; Clostridia; Clostridiales; Clostridiaceae_1; Clostridium_sensu_stricto_1* | -0.03 (0.83) | -0.09 (0.27) |
| *Actinobacteria; Coriobacteriia; Coriobacteriales; Coriobacteriaceae; Collinsella* | -0.16 (0.14) | -0.01 (0.83) |
| *Firmicutes; Erysipelotrichia; Erysipelotrichales; Erysipelotrichaceae; Coprobacillus* | -0.10 (0.22) | -0.05 (0.30) |
| *Bacteroidetes; Bacteroidia; Bacteroidales; Barnesiellaceae; Coprobacter* | 0.11 (0.28) | -0.02 (0.76) |
| *Firmicutes; Clostridia; Clostridiales; Lachnospiraceae; Coprococcus_1* | -0.11 (0.24) | 0.03 (0.60) |
| *Firmicutes; Clostridia; Clostridiales; Lachnospiraceae; Coprococcus_2* | 0.12 (0.25) | 0.09 (0.14) |
| *Firmicutes; Clostridia; Clostridiales; Lachnospiraceae; Coprococcus_3* | 0.07 (0. 43) | 0.09 (0.09) |
| *Actinobacteria; Coriobacteriia; Coriobacteriales; Coriobacteriales_Incertae_Sedis; uncultured* | -0.07 (0.47) | -0.05 (0.38) |
| *Proteobacteria; Deltaproteobacteria; Desulfovibrionales; Desulfovibrionaceae; Desulfovibrio* | -0.03 (0.70) | -0.006 (0.90) |
| *Firmicutes; Negativicutes; Selenomonadales; Veillonellaceae; Dialister* | -0.10 (0.49) | -0.14 (0.11) |
| *Firmicutes; Clostridia; Clostridiales; Lachnospiraceae; Dorea* | 0.08 (0.22) | 0.03 (0.57) |
| *Firmicutes; Clostridia; Clostridiales; Ruminococcaceae; DTU089* | 0.12 (0.17) | 0.03 (0.64) |
| *Actinobacteria; Coriobacteriia; Coriobacteriales; Eggerthellaceae; Eggerthella* | 0.03 (0.75) | -0.07 (0.21) |
| *Firmicutes; Clostridia; Clostridiales; Lachnospiraceae; Eisenbergiella* | 0.10 (0.31) | -0.03 (0.64) |
| *Proteobacteria; Gammaproteobacteria; Enterobacteriales; Enterobacteriaceae; Enterobacter* | 0.04 (0.72) | -0.14 (0.048) |
| *Firmicutes; Bacilli; Lactobacillales; Enterococcaceae; Enterococcus* | -0.05 (0.45) | -0.005 (0.90) |
| *Actinobacteria; Coriobacteriia; Coriobacteriales; Eggerthellaceae; Enterorhabdus* | 0.06 (0.59) | 0.09 (0.15) |
| *Firmicutes; Erysipelotrichia; Erysipelotrichales; Erysipelotrichaceae; Erysipelatoclostridium* | 0.13 (0.24) | -0.02 (0.76) |
| *Firmicutes; Erysipelotrichia; Erysipelotrichales; Erysipelotrichaceae; Erysipelotrichaceae_UCG-003* | 0.26 (0.09) | 0.13 (0.15) |
| *Proteobacteria; Gammaproteobacteria; Enterobacteriales; Enterobacteriaceae; Escherichia-Shigella* | 0.02 (0.88) | -0.17 (0.09) |
| *Firmicutes; Clostridia; Clostridiales; Ruminococcaceae; [Eubacterium]_coprostanoligenes group* | -0.29 (0.009) | -0.14 (0.04) |
| *Firmicutes; Clostridia; Clostridiales; Lachnospiraceae;[Eubacterium]_eligens_group* | 0.32 (0.006) | 0.15 (0.03) |
| *Firmicutes; Clostridia; Clostridiales; Lachnospiraceae; [Eubacterium]_fissicatena_group* | 0.06 (0.52) | -0.04 (0.47) |
| *Firmicutes; Clostridia; Clostridiales; Lachnospiraceae; [Eubacterium]_hallii_group* | -0.02 (0.76) | 0.004 (0.93) |
| *Firmicutes; Clostridia; Clostridiales; Lachnospiraceae; [Eubacterium]_oxidoreducens_group* | -0.15 (0.16) | -0.07 (0.27) |
| *Firmicutes; Clostridia; Clostridiales; Lachnospiraceae; [Eubacterium]_ruminantium_group* | 0.002 (0.98) | 0.11 (0.12) |
| *Firmicutes; Clostridia; Clostridiales; Lachnospiraceae; [Eubacterium]_ventriosum_group* | -0.08 (0.38) | -0.07 (0.21) |
| *Firmicutes; Clostridia; Clostridiales; Lachnospiraceae; [Eubacterium]_xylanophilum_group* | -0.10 (0.29) | 0.006 (0.92) |
| *Firmicutes; Clostridia; Clostridiales; Ruminococcaceae; Faecalibacterium* | 0.18 (0.01) | 0.06 (0.20) |
| *Firmicutes; Erysipelotrichia; Erysipelotrichales; Erysipelotrichaceae; Faecalitalea* | -0.07 (0.48) | -0.03 (0.67) |
| *Taxon=Bacteria; Firmicutes; Clostridia; Clostridiales; Family_XIII; Family_XIII_AD3011_group* | -0.12 (0.17) | 0.08 (0.11) |
| *Firmicutes; Clostridia; Clostridiales; Family_XIII; Family_XIII_UCG-001* | 0.05 (0.47) | -0.004 (0.92) |
| *Bacteroidetes; Bacteroidia; Flavobacteriales; Flavobacteriaceae; uncultured* | 0.11 (0.07) | -0.06 (0.09) |
| *Firmicutes; Clostridia; Clostridiales; Ruminococcaceae; Flavonifractor* | 0.008 (0.94) | -0.04 (0.52) |
| *Firmicutes; Clostridia; Clostridiales; Ruminococcaceae; Fournierella* | -0.10 (0.11) | -0.08 (0.03) |
| *Firmicutes; Clostridia; Clostridiales; Lachnospiraceae; Fusicatenibacter* | -0.01 (0.85) | 0.01 (0.79) |
| *Fusobacteria; Fusobacteriia; Fusobacteriales; Fusobacteriaceae; Fusobacterium* | -0.38 (0.001) | 0.02 (0.81) |
| *Cyanobacteria; Melainabacteria; Gastranaerophilales; uncultured_bacterium;Other* | 0.07 (0.40) | 0.06 (0.20) |
| *Firmicutes; Clostridia; Clostridiales; Lachnospiraceae; GCA-900066575* | -0.03 (0.71) | 0.02 (0.71) |
| *Tenericutes; Mollicutes; Mollicutes_RF39; gut_metagenome;Other* | -0.007 (0.94) | -0.02 (0.76) |
| *Proteobacteria; Gammaproteobacteria; Pasteurellales; Pasteurellaceae; Haemophilus* | 0.08 (0.41) | -0.03 (0.62) |
| *Firmicutes; Erysipelotrichia; Erysipelotrichales; Erysipelotrichaceae; Holdemanella* | -0.20 (0.17) | 0.01 (0.90) |
| *Firmicutes; Erysipelotrichia; Erysipelotrichales; Erysipelotrichaceae; Holdemania* | -0.07 (0.42) | -0.06 (0.24) |
| *Firmicutes; Clostridia; Clostridiales; Lachnospiraceae; Howardella* | -0.02 (0.75) | -0.007 (0.86) |
| *Tenericutes; Mollicutes; Mollicutes_RF39; human_gut_metagenome;Other* | 0.04 (0.57) | -0.08 (0.07) |
| *Proteobacteria; Gammaproteobacteria; Alteromonadales; Idiomarinaceae; Idiomarina* | 0.05 (0.64) | -0.01 (0.85) |
| *Firmicutes; Clostridia; Clostridiales; Ruminococcaceae; Intestinimonas* | 0.06 (0.48) | 0.02 (0.73) |
| *Tenericutes; Mollicutes; Izimaplasmatales; uncultured_organism;Other* | -0.04 (0.71) | 0.07 (0.32) |
| *Proteobacteria; Gammaproteobacteria; Enterobacteriales; Enterobacteriaceae; Klebsiella* | 0.11 (0.41) | 0.05 (0.58) |
| *Firmicutes; Clostridia; Clostridiales; Lachnospiraceae; Lachnoclostridium* | 0.04 (0.52) | -0.001 (0.97) |
| *Firmicutes; Clostridia; Clostridiales; Lachnospiraceae; Lachnospira* | 0.29 (0.01) | 0.15 (0.05) |
| *Firmicutes; Clostridia; Clostridiales; Lachnospiraceae; Lachnospiraceae_AC2044_group* | -0.01 (0.89) | 0.05 (0.44) |
| *Firmicutes; Clostridia; Clostridiales; Lachnospiraceae; Lachnospiraceae_FCS020_group* | -0.08 (0.20) | -0.03 (0.37) |
| *Firmicutes; Clostridia; Clostridiales; Lachnospiraceae; Lachnospiraceae_NC2004_group* | 0.19 (0.04) | 0.02 (0.72) |
| *Firmicutes; Clostridia; Clostridiales; Lachnospiraceae; Lachnospiraceae_ND3007_group* | 0.12 (0.25) | 0.04 (0.58) |
| *Firmicutes; Clostridia; Clostridiales; Lachnospiraceae; Lachnospiraceae_NK4A136_group* | -0.12 (0.13) | 0.08 (0.11) |
| *Firmicutes; Clostridia; Clostridiales; Lachnospiraceae; Lachnospiraceae_UCG-001* | 0.13 (0.11) | 0.03 (0.57) |
| *Firmicutes; Clostridia; Clostridiales; Lachnospiraceae; Lachnospiraceae_UCG-003* | -0.04 (0.65) | 0.12 (0.04) |
| *Firmicutes; Clostridia; Clostridiales; Lachnospiraceae; Lachnospiraceae_UCG-004* | 0.25 (0.004) | 0.03 (0.53) |
| *Firmicutes; Clostridia; Clostridiales; Lachnospiraceae; Lachnospiraceae_UCG-008* | 0.008 (0.87) | 0.04 (0.23) |
| *Firmicutes; Clostridia; Clostridiales; Lachnospiraceae; uncultured* | 0.13 (0.058) | -0.04 (0.40) |
| *Firmicutes; Clostridia; Clostridiales; Lachnospiraceae; uncultured_bacterium* | -0.09 (0.35) | -0.03 (0.67) |
| *Firmicutes; Clostridia; Clostridiales; Lachnospiraceae; Other* | 0.20 (0.005) | 0.002 (0.97) |
| *Firmicutes; Bacilli; Lactobacillales; Lactobacillaceae; Lactobacillus* | -0.06 (0.47) | -0.01 (0.81) |
| *Firmicutes; Negativicutes; Selenomonadales; Veillonellaceae; Megamonas* | 0.19 (0.09) | 0.07 (0.30) |
| *Firmicutes; Negativicutes; Selenomonadales; Veillonellaceae; Megasphaera* | -0.10 (0.24) | 0.01 (0.83) |
| *Firmicutes; Clostridia; Clostridiales; Lachnospiraceae; Moryella* | 0.02 (0.77) | -0.03 (0.60) |
| *Bacteroidetes; Bacteroidia; Bacteroidales; Muribaculaceae; gut_metagenome* | 0.02 (0.72) | -0.02 (0.60) |
| *Bacteroidetes; Bacteroidia; Bacteroidales; Muribaculaceae; uncultured_bacterium* | -0.19 (0.17) | 0.09 (0.54) |
| *Firmicutes; Clostridia; Clostridiales; Ruminococcaceae; Negativibacillus* | 0.06 (0.49) | -0.004 (0.96) |
| *Bacteroidetes; Bacteroidia; Bacteroidales; Marinifilaceae; Odoribacter* | -0.07 (0.55) | **-0.33 (<0.0001)** |
| *Firmicutes; Clostridia; Clostridiales; Ruminococcaceae; Oscillibacter* | -0.01 (0.88) | -0.06 (0.21) |
| *Firmicutes; Clostridia; Clostridiales; Ruminococcaceae; Oscillospira* | 0.04 (0.66) | -0.004 (0.94) |
| *Bacteroidetes; Bacteroidia; Bacteroidales; Tannerellaceae; Parabacteroides* | -0.15 (0.16) | -0.04 (0.53) |
| *Bacteroidetes; Bacteroidia; Bacteroidales; Prevotellaceae; Paraprevotella* | -0.20 (0.12) | 0.03 (0.67) |
| *Proteobacteria; Gammaproteobacteria; Betaproteobacteriales; Burkholderiaceae; Parasutterella* | -0.10 (0.49) | 0.05 (0.57) |
| *Firmicutes; Clostridia; Clostridiales; Peptostreptococcaceae; uncultured* | 0.11 (0.30) | 0.02 (0.81) |
| *Firmicutes; Negativicutes; Selenomonadales; Acidaminococcaceae; Phascolarctobacterium* | -0.02 (0.90) | -0.02 (0.83) |
| *Bacteroidetes; Bacteroidia; Bacteroidales; Prevotellaceae; Prevotella_1* | -0.12 (0.40) | -0.01 (0.90) |
| *Bacteroidetes; Bacteroidia; Bacteroidales; Prevotellaceae; Prevotella_2* | -0.21 (0.07) | -0.16 (0.02) |
| *Bacteroidetes; Bacteroidia; Bacteroidales; Prevotellaceae; Prevotella_7* | -0.10 (0.40) | 0.05 (0.49) |
| *Bacteroidetes; Bacteroidia; Bacteroidales; Prevotellaceae; Prevotella_9* | -0.21 (0.20) | -0.04 (0.71) |
| *Bacteroidetes; Bacteroidia; Bacteroidales; Prevotellaceae; Prevotellaceae_UCG-001* | -0.03 (0.73) | 0.06 (0.27) |
| *Bacteroidetes; Bacteroidia; Bacteroidales; Prevotellaceae; uncultured* | -0.10 (0.30) | -0.05 (0.35) |
| *Bacteroidetes; Bacteroidia; Bacteroidales; Prevotellaceae; Other* | -0.03 (0.66) | 0.06 (0.17) |
| *Verrucomicrobia; Verrucomicrobiae; Opitutales; Puniceicoccaceae; uncultured* | -0.18 (0.81) | -0.03 (0.57) |
| *Proteobacteria; Alphaproteobacteria; Rhodospirillales; uncultured; gut_metagenome* | -0.001 (0.99) | -0.07 (0.34) |
| *Proteobacteria; Alphaproteobacteria; Rhodospirillales; uncultured; uncultured_bacterium* | 0.19 (0.11) | 0.02 (0.77) |
| *Firmicutes; Clostridia; Clostridiales; Lachnospiraceae; Roseburia* | 0.04 (0.56) | -0.003 (0.94) |
| *Firmicutes; Clostridia; Clostridiales; Ruminococcaceae; Ruminiclostridium_5* | 0.005 (0.94) | 0.01 (0.76) |
| *Firmicutes; Clostridia; Clostridiales; Ruminococcaceae; Ruminiclostridium_6* | 0.07 (0.64) | 0.008 (0.93) |
| *Firmicutes; Clostridia; Clostridiales; Ruminococcaceae; Ruminiclostridium_9* | 0.15 (0.08) | 0.01 (0.79) |
| *Firmicutes; Clostridia; Clostridiales; Ruminococcaceae; Ruminococcaceae_NK4A214_group* | -0.16 (0.16) | 0.05 (0.50) |
| *Firmicutes; Clostridia; Clostridiales; Ruminococcaceae; Ruminococcaceae_UCG-002* | -0.14 (0.08) | -0.03 (0.51) |
| *Firmicutes; Clostridia; Clostridiales; Ruminococcaceae; Ruminococcaceae_UCG-003* | -0.12 (0.19) | -0.10 (0.09) |
| *Firmicutes; Clostridia; Clostridiales; Ruminococcaceae; Ruminococcaceae_UCG-004* | 0.08 (0.26) | 0.03 (0.56) |
| *Firmicutes; Clostridia; Clostridiales; Ruminococcaceae; Ruminococcaceae_UCG-005* | -0.08 (0.45) | -0.002 (0.98) |
| *Firmicutes; Clostridia; Clostridiales; Ruminococcaceae; Ruminococcaceae_UCG-010* | -0.001 (0.99) | 0.10 (0.12) |
| *Firmicutes; Clostridia; Clostridiales; Ruminococcaceae; Ruminococcaceae_UCG-011* | -0.07 (0.45) | 0.008 (0.88) |
| *Firmicutes; Clostridia; Clostridiales; Ruminococcaceae; Ruminococcaceae_UCG-013* | 0.09 (0.35) | 0.10 (0.10) |
| *Firmicutes; Clostridia; Clostridiales; Ruminococcaceae; Ruminococcaceae_UCG-014* | -0.12 (0.37) | 0.22 (0.007) |
| *Firmicutes; Clostridia; Clostridiales; Ruminococcaceae; uncultured* | 0.05 (0.54) | -0.02 (0.67) |
| *Firmicutes; Clostridia; Clostridiales; Ruminococcaceae; Ruminococcus_1* | 0.05 (0.47) | 0.09 (0.05) |
| *Firmicutes; Clostridia; Clostridiales; Ruminococcaceae; Ruminococcus_2* | 0.08 (0.49) | -0.02 (0.77) |
| *Firmicutes; Clostridia; Clostridiales; Lachnospiraceae; [Ruminococcus]_gauvreauii_group* | -0.07 (0.50) | 0.03 (0.59) |
| *Firmicutes; Clostridia; Clostridiales; Lachnospiraceae; [Ruminococcus]_gnavus_group* | 0.09 (0.46) | -0.002 (0.98) |
| *Firmicutes; Clostridia; Clostridiales; Lachnospiraceae; [Ruminococcus]_torques_group* | 0.03 (0.61) | 0.04 (0.30) |
| *Firmicutes; Clostridia; Clostridiales; Lachnospiraceae; Sellimonas* | 0.01 (0.89) | -0.03 (0.63) |
| *Actinobacteria; Coriobacteriia; Coriobacteriales; Eggerthellaceae; Senegalimassilia* | 0.11 (0.16) | 0.07 (0.19) |
| *Firmicutes; Clostridia; Clostridiales; Lachnospiraceae; Shuttleworthia* | -0.005 (0.95) | -0.09 (0.08) |
| *Actinobacteria; Coriobacteriia; Coriobacteriales; Eggerthellaceae; Slackia* | 0.02 (0.83) | 0.04 (0.34) |
| *Firmicutes; Bacilli; Lactobacillales; Streptococcaceae; Streptococcus* | 0.13 (0.29) | **0.29 (0.0003)** |
| *Firmicutes; Clostridia; Clostridiales; Ruminococcaceae; Subdoligranulum* | -0.11 (0.34) | -0.03 (0.68) |
| *Proteobacteria; Gammaproteobacteria; Betaproteobacteriales; Burkholderiaceae; Sutterella* | -0.06 (0.73) | -0.02 (0.87) |
| *Firmicutes; Erysipelotrichia; Erysipelotrichales; Erysipelotrichaceae; Turicibacter* | 0.05 (0.57) | -0.08 (0.18) |
| *Firmicutes; Clostridia; Clostridiales; Lachnospiraceae; Tyzzerella* | 0.01 (0.93) | -0.04 (0.55) |
| *Firmicutes; Clostridia; Clostridiales; Lachnospiraceae; Tyzzerella_3* | 0.12 (0.36) | -0.02 (0.79) |
| *Firmicutes; Clostridia; Clostridiales; Lachnospiraceae; Tyzzerella_4* | -0.005 (0.95) | 0.02 (0.73) |
| *Firmicutes; Clostridia; Clostridiales; Ruminococcaceae; UBA1819* | 0.05 (0.51) | 0.03 (0.62) |
| *Firmicutes; Clostridia; Clostridiales; Lachnospiraceae; UC5-1-2E3* | -0.08 (0.39) | -0.002 (0.97) |
| *Firmicutes; Negativicutes; Selenomonadales; Veillonellaceae; Veillonella* | 0.07 (0.46) | -0.13 (0.03) |
| *Lentisphaerae; Lentisphaeria; Victivallales; Victivallaceae; Victivallis* | 0.14 (0.10) | 0.11 (0.04) |
| *Firmicutes; Bacilli; Lactobacillales; Leuconostocaceae; Weissella* | 0.11 (0.09) | -0.05 (0.25) |

Abbreviations: P = Regularly consuming probiotic supplements; NP = Not consuming probiotic supplements regularly; Y = Regularly consuming yogurt; NY = Not consuming yogurt regularly.

^a^ 1,861 participants were included in the analyses: P (N=175), NP (N=1,652), 34 participants had missing data of P or NP; Y (N=818), NY (N= 1,023), 20 participants had missing data of Y or NY.

^b^ Beta coefficient (β) and P value were estimated using proc GLM adjusting for age, sex, ethnicity, antibiotic intake, smoking status, daily moderate/vigorous physical activity hours, body mass index, dietary fiber intake and total calories.

^c^ Bonferroni-corrected p-value of 0.05/152=0.00033 was applied.

**Supplemental Table 2: Association of yogurt consumption with microbiota composition (152 analyzed genera) by ethnicity in the Adiposity Phenotype Study**

|  | **Y (vs. NY)**  ---------------------------------------------------------------------------------------------------- | | | | |
| --- | --- | --- | --- | --- | --- |
| **Genera** | AA  131^a^/184^b^ | JA  162^a^/272^b^ | NH  115^a^/185^b^ | Latino  191^a^/193^b^ | White  219^a^/189^b^ |
|  | β (*P*)^cd^ | β (*P*)^cd^ | β (*P*)^cd^ | β (*P*)^cd^ | β (*P*)^cd^ |
| *Firmicutes; Negativicutes; Selenomonadales; Acidaminococcaceae; Acidaminococcus* | -0.09 (0.32) | 0.05 (0.51) | 0.03 (0.77) | -0.08 (0.28) | 0.12 (0.13) |
| *Actinobacteria; Coriobacteriia; Coriobacteriales; Eggerthellaceae; Adlercreutzia* | -0.09 (0.53) | 0.007 (0.95) | -0.13 (0.35) | -0.05 (0.66) | 0.16 (0.19) |
| *Firmicutes; Clostridia; Clostridiales; Lachnospiraceae; Agathobacter* | 0.03 (0.82) | 0.19 (0.11) | 0.12 (0.37) | 0.01 (0.91) | -0.13 (0.25) |
| *Firmicutes; Clostridia; Clostridiales; Lachnospiraceae; Agathobacter* | 0.01 (0.96) | -0.02 (0.87) | -0.27 (0.07) | -0.03 (0.82) | -0.14 (0.23) |
| *Verrucomicrobia; Verrucomicrobiae; Verrucomicrobiales; Akkermansiaceae; Akkermansia* | -0.34 (0.20) | 0.05 (0.80) | 0.15 (0.56) | -0.31 (0.15) | 0.39 (0.07) |
| *Bacteroidetes; Bacteroidia; Bacteroidales; Rikenellaceae; Alistipes* | 0.13 (0.50) | -0.28 (0.08) | 0.05 (0.79) | -0.36 (0.02) | -0.18 (0.18) |
| *Bacteroidetes; Bacteroidia; Bacteroidales; Prevotellaceae; Alloprevotella* | -0.09 (0.59) | 0.04 (0.72) | -0.18 (0.92) | -0.41 (0.02) | 0.21 (0.16) |
| *Firmicutes; Clostridia; Clostridiales; Lachnospiraceae; Anaerostipes* | 0.13 (0.23) | 0.09 (0.35) | 0.19 (0.08) | -0.11 (0.23) | 0.09 (0.34) |
| *Firmicutes; Clostridia; Clostridiales; Ruminococcaceae; Anaerotruncus* | 0.005 (0.97) | 0.12 (0.29) | -0.06 (0.69) | 0.16 (0.18) | -0.31 (0.01) |
| *Bacteroidetes; Bacteroidia; Bacteroidales; Bacteroidales_RF16_group; uncultured_bacterium* | 0.29 (0.10) | -0.28 (0.046) | -0.11 (0.59) | 0.04 (0.84) | 0.18 (0.26) |
| *Bacteroidetes; Bacteroidia; Bacteroidales; Other; Other* | -0.27 (0.07) | -0.13 (0.29) | 0.09 (0.56) | 0.08 (0.50) | 0.06 (0.64) |
| *Bacteroidetes; Bacteroidia; Bacteroidales; Bacteroidaceae; Bacteroides* | -0.17 (0.12) | 0.08 (0.27) | -0.04 (0.68) | -0.02(0.81) | -0.09 (0.29) |
| *Firmicutes; Clostridia; Clostridiales; Lachnospiraceae; [Bacteroides]_pectinophilus_group* | 0.03 (0.87) | -0.20 (0..08) | -0.004 (0.98) | -0.06 (0.65) | 0.21 (0.09) |
| *Bacteroidetes; Bacteroidia; Bacteroidales; Barnesiellaceae; Barnesiella* | -0.28 (0.24) | -0.21 (0.17) | -0.12 (0.60) | 0.22 (0.27) | 0.12 (0.52) |
| *Bacteroidetes; Bacteroidia; Bacteroidales; Barnesiellaceae; uncultured* | -0.04 (0.76) | -0.19 (0.03) | -0.05 (0.65) | 0.02 (0.86) | 0.15 (0.11) |
| *Actinobacteria; Actinobacteria; Bifidobacteriales; Bifidobacteriaceae; Bifidobacterium* | 0.002 (0.99) | -0.10 (0.50) | -0.11 (0.54) | 0.02 (0.91) | 0.17 (0.22) |
| *Proteobacteria; Deltaproteobacteria; Desulfovibrionales; Desulfovibrionaceae; Bilophila* | 0.10 (0.47) | -0.02 (0.82) | 0.03 (0.84) | -0.02 (0.87) | -0.17 (0.15) |
| *Firmicutes; Clostridia; Clostridiales; Lachnospiraceae; Blautia* | -0.03 (0.72) | 0.07 (0.31) | -0.02 (0.83) | 0.02 (0.82) | -0.03 (0.70) |
| *Firmicutes; Clostridia; Clostridiales; Ruminococcaceae; Butyricicoccus* | 0.09 (0.48) | 0.03 (0.75) | -0.01 (0.91) | -0.10 (0.32) | -0.05 (0.59) |
| *Bacteroidetes; Bacteroidia; Bacteroidales; Marinifilaceae; Butyricimonas* | -0.16 (0.28) | -0.06 (0.55) | 0.14 (0.30) | 0.04 (0.75) | 0.01 (0.86) |
| *Firmicutes; Clostridia; Clostridiales; Lachnospiraceae; Butyrivibrio* | 0.09 (0.54) | 0.23 (0.09) | 0.29 (0.07) | 0.05 (0.76) | -0.23 (0.14) |
| *Firmicutes; Clostridia; Clostridiales; Ruminococcaceae; CAG-352* | -0.13 (0.12) | 0.13 (0.37) | 0.02 (0.92) | 0.04 (0.83) | 0.11 (0.50) |
| *Firmicutes; Erysipelotrichia; Erysipelotrichales; Erysipelotrichaceae; Candidatus_Stoquefichus* | 0.20 (0.12) | 0.03 (0.77) | -0.16 (0.28) | -0.07 (0.47) | -0.02 (0.86) |
| *Firmicutes; Erysipelotrichia; Erysipelotrichales; Erysipelotrichaceae; Catenibacterium* | -0.11 (0.62) | -0.21 (0.16) | 0.13 (0.59) | -0.31 (0.15) | -0.09 (0.60) |
| *Taxon=Bacteria; Firmicutes; Clostridia; Clostridiales;Christensenellaceae; Christensenellaceae_R-7_group* | -0.11 (0.62) | -0.16 (0.28) | 0.25 (0.23) | 0.11 (0.55) | -0.05 (0.77) |
| *Synergistetes; Synergistia; Synergistales; Synergistaceae; Cloacibacillus* | -0.16 (0.24) | 0.07 (0.49) | 0.14 (0.84) | 0.21 (0.06) | 0.10 (0.37) |
| *Firmicutes; Clostridia; Clostridiales;Other;Other* | 0.006 (0.94) | -0.01 (0.75) | -0.03 (0.63) | 0.07 (0.41) | -0.02 (0.82) |
| *Firmicutes; Clostridia; Clostridiales; Clostridiales_vadinBB60_group; uncultured_bacterium* | 0.16 (0.38) | 0.19 (0.09) | -0.05 (0.76) | -0.0001 (0.99) | -0.06 (0.69) |
| *Firmicutes; Clostridia; Clostridiales; Clostridiales_vadinBB60_group; uncultured_organism* | -0.30 (0.03) | -0.07 (0.39) | 0.22 (0.08) | 0.07 (0.50) | 0.13 (0.23) |
| *Firmicutes; Clostridia; Clostridiales; Clostridiaceae_1; Clostridium_sensu_stricto_1* | -0.32 (0.14) | -0.10 (0.46) | 0.09 (0.64) | 0.14 (0.49) | -0.35 (0.06) |
| *Actinobacteria; Coriobacteriia; Coriobacteriales; Coriobacteriaceae; Collinsella* | 0.28 (0.11) | -0.14 (0.37) | -0.19 (0.25) | 0.13 (0.32) | -0.04 (0.80) |
| *Firmicutes; Erysipelotrichia; Erysipelotrichales; Erysipelotrichaceae; Coprobacillus* | 0.04 (0.75) | -0.13 (0.18) | -0.10 (0.41) | 0.008 (0.94) | -0.09 (0.40) |
| *Bacteroidetes; Bacteroidia; Bacteroidales; Barnesiellaceae; Coprobacter* | -0.05 (0.77) | -0.10 (0.37) | -0.10 (0.55) | 0.16 (0.30) | -0.07 (0.61) |
| *Firmicutes; Clostridia; Clostridiales; Lachnospiraceae; Coprococcus_1* | 0.06 (0.69) | -0.08 (0.47) | 0.04 (0.79) | -0.009 (0.95) | 0.18 (0.13) |
| *Firmicutes; Clostridia; Clostridiales; Lachnospiraceae; Coprococcus_2* | 0.31 (0.04) | -0.02 (0.86) | -0.06 (0.71) | 0.21 (0.14) | 0.11 (0.43) |
| *Firmicutes; Clostridia; Clostridiales; Lachnospiraceae; Coprococcus_3* | 0.23 (0.08) | 0.11 (0.33) | -0.14 (0.30) | 0.13 (0.24) | 0.09 (0.44) |
| *Actinobacteria; Coriobacteriia; Coriobacteriales; Coriobacteriales_Incertae_Sedis; uncultured* | 0.03 (0.85) | 0.02 (0.85) | -0.20 (0.19) | -0.06 (0.70) | -0.03 (0.83) |
| *Proteobacteria; Deltaproteobacteria; Desulfovibrionales; Desulfovibrionaceae; Desulfovibrio* | 0.13 (0.33) | -0.05 (0.59) | 0.03 (0.83) | -0.07 (0.57) | -0.04 (0.70) |
| *Firmicutes; Negativicutes; Selenomonadales; Veillonellaceae; Dialister* | -0.39 (0.13) | -0.10 (0.59) | 0.07 (0.76) | -0.33 (0.10) | -0.02 (0.92) |
| *Firmicutes; Clostridia; Clostridiales; Lachnospiraceae; Dorea* | 0.17 (0.16) | 0.10 (0.30) | 0.12 (0.25) | -0.06 (0.45) | -0.14 (0.13) |
| *Firmicutes; Clostridia; Clostridiales; Ruminococcaceae; DTU089* | -0.15 (0.30) | 0.07 (0.54) | 0.01 (0.92) | 0.06 (0.60) | -0.07 (0.55) |
| *Actinobacteria; Coriobacteriia; Coriobacteriales; Eggerthellaceae; Eggerthella* | 0.007 (0.96) | -0.11 (0.32) | -0.05 (0.70) | -0.12 (0.35) | -0.25 (0.02) |
| *Firmicutes; Clostridia; Clostridiales; Lachnospiraceae; Eisenbergiella* | 0.08 (0.60) | 0.03 (0.81) | -0.05 (0.72) | -0.12 (0.35) | -0.07 (0.57) |
| *Proteobacteria; Gammaproteobacteria; Enterobacteriales; Enterobacteriaceae; Enterobacter* | -0.23 (0.20) | 0.20 (0.16) | -0.28 (0.11) | -0.29 (0.06) | -0.16 (0.24) |
| *Firmicutes; Bacilli; Lactobacillales; Enterococcaceae; Enterococcus* | 0.08 (0.50) | -0.03 (0.63) | -0.01 (0.95) | -0.12 (0.24) | 0.09 (0.26) |
| *Actinobacteria; Coriobacteriia; Coriobacteriales; Eggerthellaceae; Enterorhabdus* | 0.32 (0.06) | -0.11 (0.45) | 0.23 (0.16) | 0.007 (0.96) | 0.12 (0.35) |
| *Firmicutes; Erysipelotrichia; Erysipelotrichales; Erysipelotrichaceae; Erysipelatoclostridium* | -0.16 (0.44) | 0.09 (0.53) | -0.05 (0.75) | -0.06 (0.68) | 0.08 (0.57) |
| *Firmicutes; Erysipelotrichia; Erysipelotrichales; Erysipelotrichaceae; Erysipelotrichaceae_UCG-003* | -0.09 (0.72) | 0.17 (0.41) | -0.03 (0.90) | 0.02 (0.92) | -0.01 (0.95) |
| *Proteobacteria; Gammaproteobacteria; Enterobacteriales; Enterobacteriaceae; Escherichia-Shigella* | -0.28 (0.13) | -0.13 (0.55) | -0.27 (0.28) | -0.16 (0.48) | -0.10 (0.58) |
| *Firmicutes; Clostridia; Clostridiales; Ruminococcaceae; [Eubacterium]_coprostanoligenes group* | -0.14 (0.43) | -0.22 (0.14) | 0.11 (0.51) | -0.32 (0.04) | -0.08 (0.58) |
| *Firmicutes; Clostridia; Clostridiales; Lachnospiraceae;[Eubacterium]_eligens_group* | -0.14 (0.43) | 0.29 (0.08) | 0.34 (0.07) | 0.26 (0.06) | 0.01 (0.94) |
| *Firmicutes; Clostridia; Clostridiales; Lachnospiraceae; [Eubacterium]_fissicatena_group* | 0.08 90.58) | -0.05 (0.64) | 0.10 (0.49) | -0.11 (0.41) | -0.11 (0.36) |
| *Firmicutes; Clostridia; Clostridiales; Lachnospiraceae; [Eubacterium]_hallii_group* | 0.10 90.39) | 0.02 (0.86) | -0.19 (0.12) | 0.25 (0.005) | -0.16 (0.11) |
| *Firmicutes; Clostridia; Clostridiales; Lachnospiraceae; [Eubacterium]_oxidoreducens_group* | -0.03 (0.85) | -0.15 (0.25) | 0.005 (0.98) | -0.08 (0.59) | -0.09 (0.52) |
| *Firmicutes; Clostridia; Clostridiales; Lachnospiraceae; [Eubacterium]_ruminantium_group* | 0.26 (0.11) | 0.09 (0.49) | -0.10 (0.57) | 0.31 (0.08) | -0.02 (0.91) |
| *Firmicutes; Clostridia; Clostridiales; Lachnospiraceae; [Eubacterium]_ventriosum_group* | -0.02 (0.92) | -0.09 (0.46) | -0.13 (0.39) | -0.15 (0.21) | 0.02 (0.85) |
| *Firmicutes; Clostridia; Clostridiales; Lachnospiraceae; [Eubacterium]_xylanophilum_group* | 0.02 (0.91) | 0.10 (0.43) | -0.15 (0.31) | -0.002 (0.99) | -0.03 (0.80) |
| *Firmicutes; Clostridia; Clostridiales; Ruminococcaceae; Faecalibacterium* | -0.02 (0.88) | 0.13 (0.22) | -0.0001 (0.99) | 0.13 (0.11) | 0.0001 (0.99) |
| *Firmicutes; Erysipelotrichia; Erysipelotrichales; Erysipelotrichaceae; Faecalitalea* | 0.17 (0.27) | -0.03 (0.77) | -0.09 (0.51) | -0.07 (0.62) | -0.08 (0.51) |
| *Taxon=Bacteria; Firmicutes; Clostridia; Clostridiales; Family_XIII; Family_XIII_AD3011_group* | 0.03 (0.81) | 0.13 (0.23) | 0.18 (0.18) | 0.05 (0.63) | 0.04 (0.68) |
| *Firmicutes; Clostridia; Clostridiales; Family_XIII; Family_XIII_UCG-001* | -0.07 (0.57) | -0.03 (0.77) | -0.04 (0.76) | 0.03 (0.78) | 0.03 (0.73) |
| *Bacteroidetes; Bacteroidia; Flavobacteriales; Flavobacteriaceae; uncultured* | -0.20 (0.04) | -0.03 (0.58) | -0.03 (0.75) | -0.04 (0.65) | -0.08 (0.35) |
| *Firmicutes; Clostridia; Clostridiales; Ruminococcaceae; Flavonifractor* | -0.11 (0.56) | 0.19 (0.13) | -0.12 (0.43) | -0.20 (0.12) | -0.003 (0.98) |
| *Firmicutes; Clostridia; Clostridiales; Ruminococcaceae; Fournierella* | -0.15 (0.08) | -0.14 (0.11) | -0.07 (0.55) | -0.01 (0.83) | -0.11 (0.20) |
| *Firmicutes; Clostridia; Clostridiales; Lachnospiraceae; Fusicatenibacter* | 0.11 (0.38) | 0.02 (0.83) | 0.07 (0.53) | -0.01 (0.91) | -0.06 (0.52) |
| *Fusobacteria; Fusobacteriia; Fusobacteriales; Fusobacteriaceae; Fusobacterium* | 0.09 (0.57) | 0.07 (0.68) | 0.07 (0.73) | -0.07 (0.62) | 0.04 (0.77) |
| *Cyanobacteria; Melainabacteria; Gastranaerophilales; uncultured_bacterium;Other* | 0.14 (0.29) | 0.07 (0.46) | 0.16 (0.23) | 0.16 (0.15) | -0.15 ( (0.18) |
| *Firmicutes; Clostridia; Clostridiales; Lachnospiraceae; GCA-900066575* | 0.15 (0.23) | 0.06 90.53) | -0.05 (0.68) | 0.01 (0.91) | -0.06 (0.59) |
| *Tenericutes; Mollicutes; Mollicutes_RF39; gut_metagenome;Other* | -0.16 (0.31) | -0.02 (0.87) | -0.13 (0.34) | 0.07 (0.64) | 0.08 (0.54) |
| *Proteobacteria; Gammaproteobacteria; Pasteurellales; Pasteurellaceae; Haemophilus* | 0.05 0.74) | -0.003 (0.98) | -0.004 (0.98) | -0.05 (0.69) | -0.09 (0.46) |
| *Firmicutes; Erysipelotrichia; Erysipelotrichales; Erysipelotrichaceae; Holdemanella* | 0.03 (0.90) | 0.11 (0.53) | 0.36 (0.16) | -0.15 (0.45) | -0.16 (0.39) |
| *Firmicutes; Erysipelotrichia; Erysipelotrichales; Erysipelotrichaceae; Holdemania* | 0.20 (0.15) | -0.09 (0.40) | -0.17 (0.22) | -0.28 (0.22) | 0.12 (0.50) |
| *Firmicutes; Clostridia; Clostridiales; Lachnospiraceae; Howardella* | -0.14 (0.17) | -0.002 (0.98) | -0.02 (0.82) | -0.03 (0.79) | 0.09 (0.32) |
| *Tenericutes; Mollicutes; Mollicutes_RF39; human_gut_metagenome;Other* | -0.11 (0.39) | -0.05 (0.45) | -0.17 (0.15) | 0.003 (0.97) | 0.10 (0.20) |
| *Proteobacteria; Gammaproteobacteria; Alteromonadales; Idiomarinaceae; Idiomarina* | -0.37 (0.03) | -0.04 (0.74) | -0.03 (0.85) | 0.04 (0.77) | 0.27 (0.035) |
| *Firmicutes; Clostridia; Clostridiales; Ruminococcaceae; Intestinimonas* | -0.07 (0.65) | 0.09 (0.41) | 0.18 (0.21) | 0.01 (0.92) | -0.10 (0.39) |
| *Tenericutes; Mollicutes; Izimaplasmatales; uncultured_organism;Other* | 0.07 (0.71) | 0.01 (0.92) | 0.05 (0.79) | -0.12 (0.50) | 0.27 (0.10) |
| *Proteobacteria; Gammaproteobacteria; Enterobacteriales; Enterobacteriaceae; Klebsiella* | 0.03 (0.89) | 0.002 (0.99) | -0.11 (0.60) | 0.15 (0.42) | 0.18 (0.27) |
| *Firmicutes; Clostridia; Clostridiales; Lachnospiraceae; Lachnoclostridium* | 0.05 (0.66) | 0.06 (0.42) | 0.10 (0.25) | -0.04 (0.65) | -0.09 (0.18) |
| *Firmicutes; Clostridia; Clostridiales; Lachnospiraceae; Lachnospira* | 0.23 (0.23) | 0.15 (0.35) | 0.19 (0.32) | 0.27(0.07) | -0.06 (0.67) |
| *Firmicutes; Clostridia; Clostridiales; Lachnospiraceae; Lachnospiraceae_AC2044_group* | -0.11 (0.49) | 0.05 (0.69) | 0.03 (0.84) | 0.14 (0.30) | -0.004 (0.98) |
| *Firmicutes; Clostridia; Clostridiales; Lachnospiraceae; Lachnospiraceae_FCS020_group* | -0.003 (0.98) | 0.12 (0.14) | -0.03 (0.75) | -0.10 (0.18) | -0.15 (0.13) |
| *Firmicutes; Clostridia; Clostridiales; Lachnospiraceae; Lachnospiraceae_NC2004_group* | -0.11 (0.45) | -0.07 (0.62) | 0.02 (0.89) | 0.07 (0.53) | 0.09 (0.46) |
| *Firmicutes; Clostridia; Clostridiales; Lachnospiraceae; Lachnospiraceae_ND3007_group* | 0.39 (0.02) | 0.03 (0.84) | 0.006 (0.97) | -0.01 (0.91) | -0.16 (0.26) |
| *Firmicutes; Clostridia; Clostridiales; Lachnospiraceae; Lachnospiraceae_NK4A136_group* | 0.22 (0.07) | 0.13 (0.22) | 0.19 (0.14) | 0.13 (0.22) | -0.17 (0.08) |
| *Firmicutes; Clostridia; Clostridiales; Lachnospiraceae; Lachnospiraceae_UCG-001* | 0.08 (0.54) | 0.04 (0.68) | -0.03 (0.82) | 0.20 (0.07) | -0.13 (0.24) |
| *Firmicutes; Clostridia; Clostridiales; Lachnospiraceae; Lachnospiraceae_UCG-003* | -0.16 (0.29) | 0.08 (0.47) | 0.06 (0.64) | 0.21 (0.11) | 0.28 (0.03) |
| *Firmicutes; Clostridia; Clostridiales; Lachnospiraceae; Lachnospiraceae_UCG-004* | -0.21 (0.16) | 0.18 (0.11) | 0.04 (0.75) | 0.10 (0.41) | 0.10 (0.36) |
| *Firmicutes; Clostridia; Clostridiales; Lachnospiraceae; Lachnospiraceae_UCG-008* | 0.01 (0.85) | 0.14 (0.036) | 0.03 (0.70) | 0.02 (0.71) | -0.009 (0.88) |
| *Firmicutes; Clostridia; Clostridiales; Lachnospiraceae; uncultured* | -0.02 (0.89) | -0.08 (0.39) | -0.07 (0.53) | 0.09 (0.28) | -0.05 (0.58) |
| *Firmicutes; Clostridia; Clostridiales; Lachnospiraceae; uncultured_bacterium* | 0.04 (0.78) | -0.15 (0.21) | -0.03 (0.88) | -0.05 (0.68) | 0.08 (0.55) |
| *Firmicutes; Clostridia; Clostridiales; Lachnospiraceae; Other* | -0.10 (0.41) | 0.15 (0.11) | -0.07 (0.56) | 0.06 (0.51) | -0.07 (0.50) |
| *Firmicutes; Bacilli; Lactobacillales; Lactobacillaceae; Lactobacillus* | -0.08 (0.60) | 0.09 (0.26) | -0.10 (0.47) | 0.02 (0.89) | -0.05 (0.59) |
| *Firmicutes; Negativicutes; Selenomonadales; Veillonellaceae; Megamonas* | -0.23 (0.14) | -0.08 (0.61) | 0.04 (0.82) | 0.34 (0.03) | 0.19 (0.16) |
| *Firmicutes; Negativicutes; Selenomonadales; Veillonellaceae; Megasphaera* | -0.06 (0.66) | 0.07 (0.44) | 0.15 (0.27) | -0,04 (0.73) | -0.07 (0.53) |
| *Firmicutes; Clostridia; Clostridiales; Lachnospiraceae; Moryella* | 0.07 (0.62) | -0.13 (0.25) | 0.007 (0.95) | -0.04 (0.67) | 0.01 (0.91) |
| *Bacteroidetes; Bacteroidia; Bacteroidales; Muribaculaceae; gut_metagenome* | -0.10 (0.25) | -0.04 (0.49) | 0.09 (0.17) | -0.07 (0.33) | 0.06 (0.37) |
| *Bacteroidetes; Bacteroidia; Bacteroidales; Muribaculaceae; uncultured_bacterium* | -0.17 (0.49) | -0.02 (0.91) | 0.19 (0.35) | 0.08 (0.67) | 0.17 (0.37) |
| *Firmicutes; Clostridia; Clostridiales; Ruminococcaceae; Negativibacillus* | -0.1 (0.95) | 0.05 (0.68) | 0.04 (0.76) | -0.10 (0.44) | 0.02 (0.82) |
| *Bacteroidetes; Bacteroidia; Bacteroidales; Marinifilaceae; Odoribacter* | -0.25 (0.16) | **-0.62 (0.0004)** | -0.24 (0.17) | -0.15 (0.29) | -0.32 (0.02) |
| *Firmicutes; Clostridia; Clostridiales; Ruminococcaceae; Oscillibacter* | -0.17 (0.22) | 0.05 (0.63) | -0.18 (0.16) | -0.11 (0.32) | 0.06 (0.57) |
| *Firmicutes; Clostridia; Clostridiales; Ruminococcaceae; Oscillospira* | -0.01 (0.94) | -0.05 (0.65) | 0.02 (0.87) | 0.02 (0.80) | -0.004 (0.97) |
| *Bacteroidetes; Bacteroidia; Bacteroidales; Tannerellaceae; Parabacteroides* | 0.04 (0.82) | -0.21 (0.16) | -0.10 (0.51) | 0.13 (0.39) | -0.01 (0.92) |
| *Bacteroidetes; Bacteroidia; Bacteroidales; Prevotellaceae; Paraprevotella* | -0.04 (0.84) | 0.02 (0.91) | -0.06 (0.76) | 0.008 (0.97) | 0.23 (0.20) |
| *Proteobacteria; Gammaproteobacteria; Betaproteobacteriales; Burkholderiaceae; Parasutterella* | -0.13 (0.59) | 0.18 (0.37) | -0.36 90.13) | 0.14 (0.46) | 0.36 (0.04) |
| *Firmicutes; Clostridia; Clostridiales; Peptostreptococcaceae; uncultured* | -0.001 (0.99) | 0.16 (0.25) | -0.17 (0.36) | 0.10 (0.51) | -0.11 (0.45) |
| *Firmicutes; Negativicutes; Selenomonadales; Acidaminococcaceae; Phascolarctobacterium* | 0.25 (0.28) | -0.04 (0.84) | -0.28 (0.20) | 0.11 (0.58) | -0.06 (0.75) |
| *Bacteroidetes; Bacteroidia; Bacteroidales; Prevotellaceae; Prevotella_1* | 0.16 (0.42) | 0.006 (0.97) | -0.22 (0.36) | -0.13 (0.56) | 0.09 (0.59) |
| *Bacteroidetes; Bacteroidia; Bacteroidales; Prevotellaceae; Prevotella_2* | -0.05 (0.82) | -0.13 (0.34) | -0.04 (0.80) | -0.38 (0.03) | -0.18 (0.26) |
| *Bacteroidetes; Bacteroidia; Bacteroidales; Prevotellaceae; Prevotella_7* | -0.001 (0.96) | 0.04 (0.77) | 0.21 (0.29) | 0.02 (0.89) | -0.008 (0.96) |
| *Bacteroidetes; Bacteroidia; Bacteroidales; Prevotellaceae; Prevotella_9* | 0.06 90.67) | -0.07 (0.71) | 0.26 (0.62) | -0.01 (0.97) | -0.04 (0.84) |
| *Bacteroidetes; Bacteroidia; Bacteroidales; Prevotellaceae; Prevotellaceae_UCG-001* | 0.06 (0.73) | 0.08 (0.52) | 0.13 (0.37) | 0.05 (0.69) | 0.03 (0.82) |
| *Bacteroidetes; Bacteroidia; Bacteroidales; Prevotellaceae; uncultured* | 0.06 (0.73) | -0.16 (0.16) | 0.03 (0.82) | -0.11 (0.44) | -0.06 (0.62) |
| *Bacteroidetes; Bacteroidia; Bacteroidales; Prevotellaceae; Other* | -0.18 (0.14) | 0.06 (0.48) | 0.03 (0.77) | 0.06 (0.61) | 0.25 (0.01) |
| *Verrucomicrobia; Verrucomicrobiae; Opitutales; Puniceicoccaceae; uncultured* | 0.001 (0.99) | 0.03 (0.65) | -0.05 (0.70) | 0.008 (0.94) | -0.14 (0.15) |
| *Proteobacteria; Alphaproteobacteria; Rhodospirillales; uncultured; gut_metagenome* | 0.08 (0.68) | -0.21 (0.12) | -0.11 (0.59) | 0.13 (0.43) | -0.17 (0.30) |
| *Proteobacteria; Alphaproteobacteria; Rhodospirillales; uncultured; uncultured_bacterium* | -0.21 (0.22) | -0.11 (0.47) | 0.28 (0.15) | 0.15 (0.37) | 0.03 (0.84) |
| *Firmicutes; Clostridia; Clostridiales; Lachnospiraceae; Roseburia* | 0.06 (0.60) | 0.09 (0.36) | 0.04 (0.72) | -0.02 (0.84) | -0.03 (0.76) |
| *Firmicutes; Clostridia; Clostridiales; Ruminococcaceae; Ruminiclostridium_5* | 0.11 (0.27) | -0.03 (0.70) | 0.10 (0.80) | 0.10 (0.28) | 0.09 (0.76) |
| *Firmicutes; Clostridia; Clostridiales; Ruminococcaceae; Ruminiclostridium_6* | -0.02 (0.95) | -0.09 (0.54) | 0.22 (0.32) | -0.12 (0.59) | 0.02 (0.91) |
| *Firmicutes; Clostridia; Clostridiales; Ruminococcaceae; Ruminiclostridium_9* | -0.09 (0.53) | 0.06 (0.60) | 0.14 (0.26) | 0.04 (0.76) | 0.09 (0.34) |
| *Firmicutes; Clostridia; Clostridiales; Ruminococcaceae; Ruminococcaceae_NK4A214_group* | 0.03 (0.87) | 0.08 (0.54) | 0.20 (0.25) | -0.04 (0.81) | -0.04 (0.80) |
| *Firmicutes; Clostridia; Clostridiales; Ruminococcaceae; Ruminococcaceae_UCG-002* | -0.05 (0.72) | -0.09 (0.43) | 0.16 (0.25) | -0.11 (0.29) | -0.01 (0.91) |
| *Firmicutes; Clostridia; Clostridiales; Ruminococcaceae; Ruminococcaceae_UCG-003* | -0.17 (0.29) | -0.08 (0.47) | -0.03 (0.84) | -0.01 (0.91) | 0.25 (0.037) |
| *Firmicutes; Clostridia; Clostridiales; Ruminococcaceae; Ruminococcaceae_UCG-004* | 0.05 (0.64) | 0.05 (0.60) | -0.02 (0.81) | 0.03 (0.77) | 0.03 (0.71) |
| *Firmicutes; Clostridia; Clostridiales; Ruminococcaceae; Ruminococcaceae_UCG-005* | -0.007 (0.97) | -0.20 (0.16) | 0.13 (0.44) | 0.003 (0.98) | 0.05 (0.72) |
| *Firmicutes; Clostridia; Clostridiales; Ruminococcaceae; Ruminococcaceae_UCG-010* | -0.27 (0.12) | 0.23 (0.07) | 0.14 (0.37) | 0.14 (0.35) | 0.10 (0.47) |
| *Firmicutes; Clostridia; Clostridiales; Ruminococcaceae; Ruminococcaceae_UCG-011* | -0.04 (0.79) | -0.02 (0.83) | -0.20 (0.15) | 0.03 (0.80) | 0.20 (0.08) |
| *Firmicutes; Clostridia; Clostridiales; Ruminococcaceae; Ruminococcaceae_UCG-013* | 0.40 (0.02) | -0.09 (0.48) | 0.18 (0.23) | -0.06 (0.63) | 0.23 (0.06) |
| *Firmicutes; Clostridia; Clostridiales; Ruminococcaceae; Ruminococcaceae_UCG-014* | -0.03 (0.89) | 0.08 (0.59) | 0.07 (0.73) | 0.41 (0.035) | 0.44 (0.02) |
| *Firmicutes; Clostridia; Clostridiales; Ruminococcaceae; uncultured* | -0.03 (0.82) | -0.09 (0.42) | -0.06 (0.67) | 0.006 (0.95) | 0.23 (0.03) |
| *Firmicutes; Clostridia; Clostridiales; Ruminococcaceae; Ruminococcus_1* | 0.02 (0.87) | 0.14 (0.24) | 0.09 (0.43) | -0.14 (0.37) | 0.16 (0.07) |
| *Firmicutes; Clostridia; Clostridiales; Ruminococcaceae; Ruminococcus_2* | -0.008 (0.97) | -0.05 (0.76) | 0.09 (0.66) | -0.03 (0.37) | 0.01 (0.95) |
| *Firmicutes; Clostridia; Clostridiales; Lachnospiraceae; [Ruminococcus]_gauvreauii_group* | 0.40 (0.01) | -0.19 (0.15) | 0.26 (0.09) | -0.03 (0.82) | -0.08 (0.52) |
| *Firmicutes; Clostridia; Clostridiales; Lachnospiraceae; [Ruminococcus]_gnavus_group* | -0.02 (0.91) | 0.35 (0.036) | -0.29 (0.10) | -0.02 (0.91) | -0.12 (0.39) |
| *Firmicutes; Clostridia; Clostridiales; Lachnospiraceae; [Ruminococcus]_torques_group* | 0.11 (0.28) | 0.10 (0.27) | 0.08 (0.42) | -0.01 (0.84) | -0.04 (0.52) |
| *Firmicutes; Clostridia; Clostridiales; Lachnospiraceae; Sellimonas* | 0.05 (0.74) | -0.21 (0.07) | 0.01 (0.94) | -0.07 (0.54) | 0.12 90.23) |
| *Actinobacteria; Coriobacteriia; Coriobacteriales; Eggerthellaceae; Senegalimassilia* | -0.10 (0.44) | 0.05 (0.56) | -0.02 (0.88) | 0.22 (0.05) | 0.06 (0.58) |
| *Firmicutes; Clostridia; Clostridiales; Lachnospiraceae; Shuttleworthia* | -0.17 (0.25) | -0.04 (0.72) | 0.02 (0.88) | -0.12 (0.30) | -0.11 (0.31) |
| *Actinobacteria; Coriobacteriia; Coriobacteriales; Eggerthellaceae; Slackia* | -0.02 (0.88) | 0.06 (0.44) | 0.12 (0.35) | 0.10 (0.37) | -0.05 (0.62) |
| *Firmicutes; Bacilli; Lactobacillales; Streptococcaceae; Streptococcus* | 0.34 (0.09) | **0.56 (0.0009)** | 0.17 (0.38) | 0.18 (0.32) | 0.15 (0.32) |
| *Firmicutes; Clostridia; Clostridiales; Ruminococcaceae; Subdoligranulum* | 0.05 (0.79) | 0.02 (0.91) | -0.06 (0.72) | -0.05 (0.64) | -0.11 (0.45) |
| *Proteobacteria; Gammaproteobacteria; Betaproteobacteriales; Burkholderiaceae; Sutterella* | 0.72 (0.006) | -0.24 (0.25) | 0.43 (0.07) | -0.36 (0.09) | -0.32 (0.10) |
| *Firmicutes; Erysipelotrichia; Erysipelotrichales; Erysipelotrichaceae; Turicibacter* | -0.28 (0.08) | -0.07 (0.53) | 0.03 (0.83) | -0.13 (0.33) | -0.03 (0.81) |
| *Firmicutes; Clostridia; Clostridiales; Lachnospiraceae; Tyzzerella* | -0.04 (0.83) | -0.09 (0.56) | 0.02 (0.92) | 0.01 (0.94) | -0.07 (0.64) |
| *Firmicutes; Clostridia; Clostridiales; Lachnospiraceae; Tyzzerella_3* | 0.04 (0.84) | 0.12 (0.49) | -0.25 (0.22) | -0.18 (0.28) | 0.15 (0.33) |
| *Firmicutes; Clostridia; Clostridiales; Lachnospiraceae; Tyzzerella_4* | 0.05 (0.71) | 0.01 (0.89) | -0.07 (0.63) | 0.08 (0.51) | -0.01 (0.91) |
| *Firmicutes; Clostridia; Clostridiales; Ruminococcaceae; UBA1819* | -0.01 (0.92) | 0.17 (0.11) | -0.06 (0.65) | 0.06 (0.60) | -0.02 (0.87) |
| *Firmicutes; Clostridia; Clostridiales; Lachnospiraceae; UC5-1-2E3* | -0.19 (0.20) | 0.13 (0.24) | -0.13 (0.40) | 0.08 (0.53) | 0.02 (0.89) |
| *Firmicutes; Negativicutes; Selenomonadales; Veillonellaceae; Veillonella* | 0.02 (0.89) | -0.14 (0.29) | -0.19 (0.20) | 0.13 (0.96) | -0.25 (0.04) |
| *Lentisphaerae; Lentisphaeria; Victivallales; Victivallaceae; Victivallis* | 0.22 (0.10) | 0.13 (0.15) | -0.03 (0.81) | 0.05 (0.70) | 0.20 (0.08) |
| *Firmicutes; Bacilli; Lactobacillales; Leuconostocaceae; Weissella* | 0.01 (0.90) | -0.09 (0.24) | -0.15 (0.22) | 0.03 (0.72) | -0.03 (0.72) |
| **OTUs** |  |  |  |  |  |
| *Streptococcus; Uncultured Bacterium* | 0.48 (0.02) | **0.70 (<0.0001)** | 0.27 (0.15) | 0.15 (0.46) | 0.34 (0.04) |

Abbreviations: Y = Regularly consuming yogurt; NY = Not consuming yogurt regularly; AA = African American; JA = Japanese American; NH = Native Hawaiian.

^a^ Number of participants with regular yogurt consumption (Y).

^b^ Number of participants without regular yogurt consumption (NY).

^c^ Beta coefficient (β) and P value were estimated using proc GLM adjusting for age, sex, ethnicity, antibiotic intake, smoking status, daily moderate/vigorous physical activity hours, body mass index, dietary fiber intake and total calories.

^d^ Bonferroni-corrected p-value of 0.05/152=0.00033 was applied.
